# Multiple Lineages of Nematode-*Wolbachia* Symbiosis in Supergroup F and Convergent Loss of Bacterioferritin in Filarial *Wolbachia*

**DOI:** 10.1093/gbe/evad073

**Published:** 2023-05-08

**Authors:** Amit Sinha, Zhiru Li, Catherine B Poole, Laurence Ettwiller, Nathália F Lima, Marcelo U Ferreira, Fanny F Fombad, Samuel Wanji, Clotilde K S Carlow

**Affiliations:** New England Biolabs, Ipswich, Massachusetts 01938; New England Biolabs, Ipswich, Massachusetts 01938; New England Biolabs, Ipswich, Massachusetts 01938; New England Biolabs, Ipswich, Massachusetts 01938; Department of Parasitology, Institute of Biomedical Sciences, University of São Paulo, Brazil; Department of Parasitology, Institute of Biomedical Sciences, University of São Paulo, Brazil; Department of Microbiology and Parasitology, University of Buea, Cameroon; Department of Microbiology and Parasitology, University of Buea, Cameroon; New England Biolabs, Ipswich, Massachusetts 01938

**Keywords:** *Wolbachia*, filaria, evolution, bacterioferritin, heme, phylogenomics

## Abstract

The intracellular endosymbiotic proteobacteria *Wolbachia* have evolved across the phyla nematoda and arthropoda. In *Wolbachia* phylogeny, supergroup F is the only clade known so far with members from both arthropod and filarial nematode hosts and therefore can provide unique insights into their evolution and biology. In this study, four new supergroup F *Wolbachia* genomes have been assembled using a metagenomic assembly and binning approach, *w*Moz and *w*Mpe from the human filarial parasites *Mansonella ozzardi* and *M. perstans*, and *w*Ocae and *w*MoviF from the blue mason bee *Osmia caerulescens* and the sheep ked *Melophagus ovinus*, respectively. A comprehensive phylogenomic analysis revealed two distinct lineages of filarial *Wolbachia* in supergroup F, indicating multiple horizontal transfer events between arthropod and nematode hosts. The analysis also reveals that the evolution of *Wolbachia*-filaria symbioses is accompanied by a convergent pseudogenization and loss of the bacterioferritin gene, a phenomenon found to be shared by all filarial *Wolbachia*, even those outside supergroup F. These observations indicate that differences in heme metabolism might be a key feature distinguishing filarial and arthropod *Wolbachia*. The new genomes provide a valuable resource for further studies on symbiosis, evolution, and the discovery of new antibiotics to treat mansonellosis.

**Significance**

*Wolbachia* are bacterial endosymbionts of medically important parasitic filarial nematodes and arthropods. The evolutionary history and biological roles of *Wolbachia* in these different hosts are not well understood. The supergroup F in *Wolbachia* phylogeny harbors members from both filarial and arthropod hosts, providing an unparalleled opportunity for genomic comparisons to uncover distinguishing characteristics. This study provides new genomes from filarial and arthropod *Wolbachia* from this unique supergroup. Their phylogenomic analysis reveals multiple, independent transfers of *Wolbachia* between arthropod and filarial hosts. Remarkably, filaria–*Wolbachia* associations are associated with a convergent loss of the bacterioferritin gene, a key regulator of heme metabolism. Heme supplementation is considered a critical component of *Wolbachia*–filaria symbiosis. We demonstrate bacterioferritin loss is a novel feature exclusive to filarial *Wolbachia*.

## Introduction

*Wolbachia* are Gram-negative α-proteobacteria of the order *Rickettsiales*, present as intracellular symbionts in many species of parasitic filarial nematodes and arthropods (Werren et al. 2008). Although the *Wolbachia* associations in arthropods range from reproductive parasites to nutritional mutualists (Werren et al. 2008; Zug and Hammerstein 2015), in filarial nematodes, *Wolbachia* is an obligate mutualist (Taylor et al. 2005) and essential for worm development, fecundity, and survival (Taylor et al. 2005; Pfarr and Hoerauf 2006). *w*Mpe and *w*Moz are endosymbionts of *Mansonella perstans* and *M. ozzardi*, respectively, the human filarial parasites responsible for mansonellosis. Mansonellosis is the most prevalent of all human filariases, yet the least studied and most neglected (Downes and Jacobsen 2010; Simonsen et al. 2011; Lima et al. 2016; Mediannikov and Ranque 2018; Ta-Tang et al. 2018; Bélard and Gehringer 2021; Sandri et al. 2021). *M. perstans* is considered to be the most common filaria in West and Central Africa (Simonsen et al. 2011; Ta-Tang et al. 2018) and is also found in the Amazon rainforest and the Caribbean coast of South America (Tavares da Silva et al. 2017; Ta-Tang et al. 2018). *M. ozzardi* infections have been reported from many countries in South and Central America, including some Caribbean Islands (Lima et al. 2016; Raccurt 2018; Ta-Tang et al. 2018; Calvopina et al. 2019). Coinfections of *M. perstans* and *M. ozzardi* are also known to exist in South America (Kozek et al. 1983; Crainey et al. 2020), complicating treatment as the two species respond differently to the commonly used antifilarial drugs (Ta-Tang et al. 2018). Antibiotics targeting *Wolbachia* have been successful in treating human filarial diseases such as onchocerciasis and lymphatic filariasis (Pfarr and Hoerauf 2006; Langworthy et al. 2000; Bazzocchi et al. 2008; Clare et al. 2019; Taylor et al. 2019; Hübner et al. 2019; Hong et al. 2019, 10). Doxycycline has been shown to clear *M. perstans* microfilariae (Keiser et al. 2008; Coulibaly et al. 2009; Ta-Tang et al. 2018) and may have some efficacy against *M. ozzardi*, as well as dual infections with *M. ozzardi* and *M. perstans* (Crainey et al. 2020); however, no such clinical trials have been reported.

The *Wolbachia* present in the filarial nematodes of the genus *Mansonella* have a unique phylogenetic position compared with *Wolbachia* present in other human filarial parasites. The latest phylogenetic classification, based on multi-locus sequence typing (MLST), assigns *Wolbachia* to various monophyletic supergroups (Lefoulon et al. 2016; Lefoulon, Clark, Guerrero, et al. 2020a; Lefoulon, Clark, Borveto, et al. 2020b). Supergroups A, B, E, H, and S are comprised entirely of arthropod *Wolbachia*, whereas supergroups C, D, and J consist of filarial *Wolbachia* exclusively. Interestingly, the *Wolbachia* from *Mansonella* are classified under supergroup F, the only supergroup currently known to have *Wolbachia* members from filarial nematode as well as arthropod hosts. There is considerable interest in studying these *Wolbachia* to better understand the evolution and biology of arthropod and filarial *Wolbachia* (Hosokawa et al. 2010; Nikoh et al. 2014; Lefoulon et al. 2016; Lefoulon, Clark, Guerrero, et al. 2020a).

Genomic information provides a fundamental resource for these investigations, yet only two genomes from supergroup F are currently available in public databases, namely the genomes of *Wolbachia w*Cle from the bedbug *Cimex lectularius* (Nikoh et al. 2014), and *w*Mhie, from *Madathamugadia hiepei*, a filarial parasite of geckos (Lefoulon, Clark, Guerrero, et al. 2020a). In the current study, the genomes of supergroup F *Wolbachia*, *w*Moz and *w*Mpe from human filarial parasites *M. ozzardi* and *M. perstans* respectively, are assembled and analyzed. The genomes of arthropod-associated supergroup F *Wolbachia*, *w*Ocae and *w*MoviF, from the mason bee, *Osmia caerulescens,* and sheep ked, *Melophagus ovinus*, are also reported. It is currently not known whether the *Wolbachia* of *M. ovinus* or *O. caerulescens* are facultative or obligate symbionts, or if they induce cytoplasmic incompatibility in these hosts. Genome-wide analyses of synteny, and annotations and comparative analysis of phage-derived gene content, cytoplasmic incompatibility genes and metabolic pathways of these genomes are described. In addition, comprehensive phylogenomic analyses of these four new genomes and all the available *Wolbachia* genomes were performed to generate comprehensive and robust phylogenetic trees and confirmed the position of the new genomes within supergroup F. These analyses revealed at least two distinct clades within supergroup F, each containing *Wolbachia* members from filarial as well as arthropod hosts, indicating multiple horizontal transfers between the two host phyla. Interestingly, the bacterioferritin gene was found to be pseudogenized in both clades of filarial *Wolbachia* in supergroup F, but not in their closest arthropod *Wolbachia*. The loss of bacterioferritin was discovered to be a feature common to all filarial *Wolbachia*.

## Results

### Genomes of *w*Moz and *w*Mpe, Supergroup F *Wolbachia* From Human Filarial Parasites *M. ozzardi* and *M. perstans*

A metagenomic assembly and binning pipeline suitable for complex clinical samples were utilized to analyze sequencing data from four *Mansonella* isolates. The binning of assembled metagenome scaffolds using BlobTools identified distinct clusters of scaffolds (“blobs”) corresponding to the *Wolbachia* genome, distinguishable from blobs corresponding to the genomes of the worm hosts and associated microbiota.

For the *M. ozzardi* isolate Moz1 from Brazil, only fragments of *Wolbachia* genome at low coverage were obtained and were not analyzed further (supplementary fig. S1*a*, Supplementary Material online). The BlobTools analysis of *M. ozzardi* isolate Moz2 from Venezuela (supplementary fig. S1*b*, Supplementary Material online) yielded a draft *w*Moz assembly of 1,073,310 bp in size comprised of 93 scaffolds. The N50 size of this assembly is 17.225 kb, and the largest scaffold is 37.481 kb in length (table 1). Of the two *M. perstans* isolates Mpe1 and Mpe2 from Cameroon, only Mpe1 yielded a *Wolbachia* assembly (supplementary fig. S2, Supplementary Material online). The *w*Mpe assembly is comprised of 1,058,123 bp in 170 scaffolds, with a N50 size of 10.041 kb and the largest scaffold 28.485 kb in length (table 1).

**Table 1:** Characteristics of Genomes of *w*Moz and *w*Mpe and Other *Wolbachia* From the Supergroup F

	*w*Moz	*w*Mpe	*w*MoviF	*w*Ocae	*w*Mhie	*w*Cle
Assembled genome size	1,073,310 bp	1,058,123 bp	1,008,858 bp	1,201,397 bp	1,025,329 bp	1,250,060 bp
Number of scaffolds	93	170	196	186	208	1
Largest scaffold	37.481 kb	28.485 kb	20.403 kb	21.794 kb	21.863 kb	-
Scaffold N50 size	17.225 kb	10.041 kb	7.443 kb	8.755 kb	6.845 kb	-
%GC	35.64	35.37	36.20	36.06	36.09	36.25
Predicted genes	1,079	1,058	1,009	1,173	1,081	1,246
Predicted proteins	888	864	928	1,055	907	981
Predicted pseudogenes	145	153	37	77	137	224
tRNA genes	36	34	35	34	30	34
BUSCO score (%)	78.1	75.3	81.3	80.4	79.0	81.7
Input reads	This study	This study	ERR969522 Nováková et al. 2015	SRR122170 Gerth et al. 2014	Lefoulon et al. 2020a, 2020b	Nikoh et al. 2014
Assembly accession and reference	GCF_020278625.1 This study	GCF_020278605.1 This study	GCA_023661065.1 This study	GCA_023661085.1 This study	GCF_013366855.1 Lefoulon et al. 2020a, 2020b	GCF_000829315.1 Nikoh et al. 2014

**Fig. 1.**
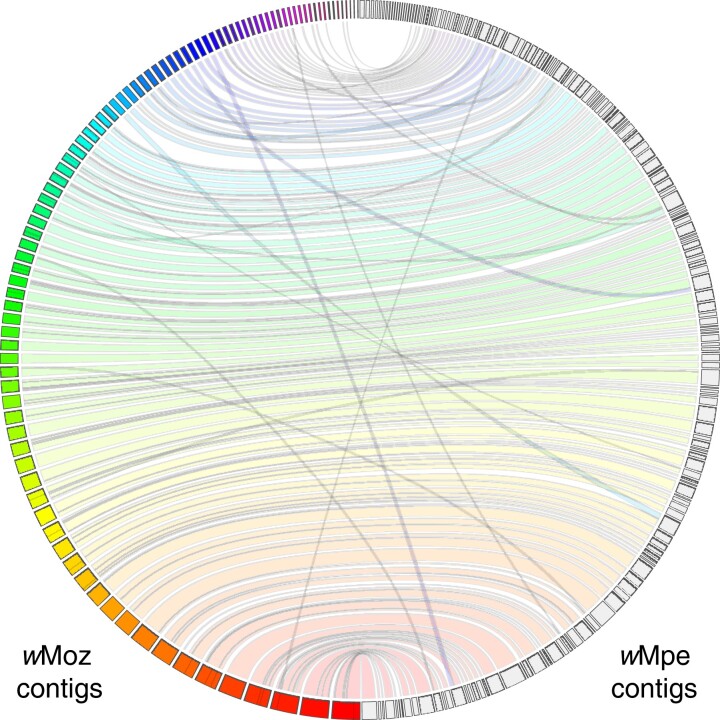
High-sequence similarity and synteny between the *de novo* assembled *w*Moz and *w*Mpe genomes. The *Wolbachia* genomes assembled from two biologically and geographically distinct isolates display a high level of synteny and intergenome consistency. The genomes were aligned using minimap2 and visualized using the JupiterPlot software. The *w*Moz scaffolds are displayed as colored boxes in the left semi-circle, whereas the *w*Mpe scaffolds are shown as gray boxes in the right semicircle. The syntenic regions are marked by bands connecting scaffolds from one assembly to the corresponding region in the other assembly. Regions of rearrangement are shown as bands crisscrossing the horizontal bands of the syntenic regions.

Whole genome alignment of *w*Moz and *w*Mpe demonstrated high sequence similarity and colinearity of these independently assembled and closely related *Wolbachia* (fig. 1). A comparison was also made to the most closely related and the only complete genome available in the Clade F supergroup, namely *w*Cle, where high sequence synteny and colinearity were also observed (supplementary fig. S3, Supplementary Material online).

Gene prediction using the NCBI PGAP pipeline identified 888 protein coding genes in *w*Moz and 864 protein coding genes in *w*Mpe. A BUSCO analysis (v5.1.3) was performed on these proteins to check for presence of the 219 genes conserved across most proteobacteria (“proteobacteria_odb10” database in BUSCO). The BUSCO scores of *w*Moz and *w*Mpe were 77.6% and 74.4%, respectively (table 1). These scores are typical for *Wolbachia,* even with complete genomes. For example, the BUSCO scores for *Wolbachia w*Ov from the human filarial parasite *Onchocerca volvulus*, *w*Oo from the bovine parasite *On. ochengi*, and *w*Bm from the human filarial parasite *Brugia malayi* are 76.7%, 75.8%, and 79.5%, respectively (supplementary fig. S4, Supplementary Material online). Within supergroup F, the BUSCO score for filarial *Wolbachia w*Mhie is 79%, and it is 81.7% for the arthropod *Wolbachia w*Cle.

### Different Isolates of *Mansonella* Harbor Varying Levels of *Wolbachia*

The metagenomic assembly and binning approach enabled the determination of relative amounts of *Wolbachia* in different isolates by comparing read coverages of the *Mansonella-* and *Wolbachia-*specific scaffolds in the metagenomic assemblies (table 2). In the *M. ozzardi* sample Moz2, the median coverage of scaffolds classified as *Mansonella* was 360 × , whereas that of the *Wolbachia* scaffolds was 13 × . Assuming 1,000 nematode cells per microfilaria (Basyoni and Rizk 2016), each parasite is estimated to harbor 37 *Wolbachia* cells. For the other *M. ozzardi* isolate Moz1, the median coverage for nematode scaffolds was 57 × , and the corresponding *Wolbachia* coverage was only 3X. Therefore, titer calculations were not performed for the Moz1 isolate as they would not be robust at such low coverage and incomplete assembly. For the *M. perstans* sample (Mpe1), the median coverage of nematode scaffolds and *Wolbachia* scaffolds were 1,032 × and 30 × respectively, yielding an estimated titer of 30 *Wolbachia* per microfilaria. In the other *M. perstans* isolate Mpe2, even though the median coverage for nematode scaffolds is relatively high at 633X, only a very small portion of the *Wolbachia* genome, 15 kb in 12 scaffolds (less than 2% of 1.073 Mb *w*Mpe1 genome) with a median coverage of 7 × could be obtained during the metagenomic assembly, indicating a very low titer of *Wolbachia* in this isolate. The low titer was also confirmed using a complementary analysis, namely mapping the Mpe2 reads against the *w*Mpe1 assembly. In this analysis, the median depth of coverage obtained was only 9 × , whereas the breadth of coverage (number of bases covered at 1 × depth of coverage) was observed to be 83 kb only, just 8% of the *w*Mpe1 genome. Because both isolates were collected in Cameroon, these observations point to variation in *Wolbachia* titers within the same species in a geographical area and could explain the conflicting reports of PCR-based detection of *Wolbachia* in *M. perstans* (Grobusch et al. 2003; Keiser et al. 2008; Coulibaly et al. 2009; Gehringer et al. 2014; Debrah et al. 2019; Sandri et al. 2021).

**Table 2:** Estimation of *Wolbachia* Levels per Microfilaria

Host species	Isolate name	Isolate Location	Median read coverage of *Mansonella* scaffolds (M)	Number and total size of assembled *Wolbachia* scaffolds	Number and total size of assembled *Wolbachia* scaffolds	*Wolbachia* genome assembled	Median read coverage of *Wolbachia* scaffolds (W)	Number of *Wolbachia* per microfilaria^a^ (1000×W/M)
*M. ozzardi*	Moz1	Brazil	57	173 scaffolds	0.12 Mb	No	3	NA**^b^**
*M. ozzardi*	Moz2	Venezuela	360	93 scaffolds	1.073 Mb	*w*Moz	13	37
*M. perstans*	Mpe1	Cameroon	1,032	170 scaffolds	1.058 Mb	*w*Mpe	30	30
*M. perstans*	Mpe2	Cameroon	633	5 scaffolds	8.781 kb	No	NA**^b^**	NA**^b^**

a Assuming 1000 cells per microfilaria.

b Not calculated due to the very low number or coverage of *Wolbachia* scaffolds.

### Genomes of *w*Ocae and *w*MoviF, Supergroup F *Wolbachia* From the Arthropods *O. caerulescens* and *M. ovinus*

For the *Wolbachia w*Ocae from the mason bee *O. caerulescens*, metagenomic assembly and binning were performed on Illumina sequencing reads available from a previous study (Gerth et al. 2014). A BlobTools analysis of the assembled scaffolds identified a distinct cluster of 186 *Wolbachia* scaffolds (supplementary fig. S5*a*, Supplementary Material online) with a total size of 1.2 Mb. A whole-genome alignment of these *w*Ocae contigs against the *w*Cle demonstrated high quality of this assembly (supplementary fig. S5*b*, Supplementary Material online). The NCBI-PGAP pipeline identified 899 protein coding genes, which had a BUSCO score of 79% (table 1). During the preparation of this manuscript, a metagenomic assembly WOLB0012 of *Wolbachia* from the same raw data was published (Scholz et al. 2020), but only the unannotated nucleotide sequence is available in NCBI database. A BlobPlot analysis of this assembly indicated the presence of arthropod contigs (supplementary fig. S6, Supplementary Material online). Therefore, instead of the WOLB0012 assembly, the carefully curated and annotated version of the *w*Ocae assembly described in this manuscript was used for all subsequent analyses.

The *Wolbachia* genome from the sheep ked *M. ovinus* was obtained from analysis of publicly available reads. A first round of metagenomic assembly and BlobTools analysis revealed a complex mixture of multiple *Wolbachia* as well as other proteobacterial endosymbionts (supplementary fig. S7, Supplementary Material online). The total size of the 693 *Wolbachia* scaffolds identified in this analysis was 1.6 Mb, substantially larger than any *Wolbachia* genomes known so far. In addition, these scaffolds were spread over an unusually wide coverage range, over two orders of magnitude. A manual inspection of the blastn hits of this assembly revealed that some scaffolds had better hits against the genome of supergroup F *Wolbachia w*Cle, whereas others were more similar to the supergroup A *Wolbachia w*Mel or supergroup B *Wolbachia w*AlbB. Together, these observations point to multiple *Wolbachia* genomes in this sample. This complexity necessitated an iterative mapping and assembly approach utilizing only the reads that mapped to *Wolbachia* genomes, which produced 572 *Wolbachia* scaffolds with a total size 1.48 Mb (supplementary fig. S8*a*, Supplementary Material online). To identify the correct supergroup for each scaffold, its sequence similarity and query coverage in comparison with the *w*Cle, *w*Mel, or *w*AlbB genomes were analyzed. The median percentage identity as well as query-coverage scores was observed to be the highest against the *w*Cle genome, followed by the *w*Mel genomes, and was much lower when compared with the *w*AlbB genome (supplementary fig. S8*b*and*c*, Supplementary Material online). Therefore, to isolate the supergroup F scaffolds, alignments of each scaffold to *w*Cle genome was compared with its alignments against the *w*Mel genome. Among the 572 scaffolds, 40 scaffolds had a nucmer hit only to the *w*Cle genome and were therefore included in the supergroup F bin, 214 scaffolds had a hit only to the *w*Mel genome (supplementary fig. S8*d*, Supplementary Material online) and were included in the supergroup A bin, whereas 84 scaffolds that did not have a hit in either *w*Cle of *w*Mel genome were found to be more similar to other *Wolbachia* from supergroups A or B. For the 234 scaffolds which had a nucmer hit to both *w*Cle and *w*Mel genomes, an affiliation score, calculated as the difference between its percentage identity to the *w*Cle genome and its percentage identity to the *w*Mel genome, was analyzed. This score showed a clear bimodal distribution, with a range from −15 to 13 (supplementary fig. S8*e*, Supplementary Material online), confirming the presence of two *Wolbachia* from different supergroups in the host *M. ovinus*. The scaffolds which had an affiliation score greater than zero were assigned to the supergroup F. These 156 scaffolds also showed a sequencing read-coverage distribution similar to the 40 scaffolds that had exclusive hits to the *w*Cle genome (supplementary fig. S8*f*, Supplementary Material online), confirming their classification into supergroup F. Finally, the classification of contigs as supergroup F was validated through an additional blobplot analysis using Illumina reads from a new metagenomic sequencing of a *M. ovinus* sample from China (Zhang et al. 2023). A combination of sequencing coverage from this new analysis and the affiliation scores described above achieved a clear classification (supplementary fig. S8*g*, Supplementary Material online). A whole-genome alignment of the contigs classified as *w*MoviF against the *w*Cle demonstrated high quality of this assembly (supplementary fig. S8*h*, Supplementary Material online). The final supergroup F bin comprised of 196 scaffolds with a total size of 1.01 Mb was designated as the genome for *Wolbachia w*MoviF, where the suffix F denotes the supergroup. NCBI-PGAP annotations identified 928 protein coding genes, which had a BUSCO score of 81.3% (table 1) and no duplicated core genes (supplementary fig. S4, Supplementary Material online). The metagenomic bin for the putative supergroup A *Wolbachia* was highly fragmented (301 scaffolds, median length = 823 bp, total size = 0.387 Mb) and was not analyzed further. Recently, a metagenomic assembly of *Wolbachia* (GenBank accession WOLB1015), based on the same raw reads, became available (Scholz et al. 2020). However, multiple analyses demonstrate that this assembly is a mixture of two different *Wolbachia*. First, the protein coding genes in WOLB1015 were predicted using PROKKA, and their BUSCO score was calculated. This analysis revealed duplications of 17% of single copy orthologs (SCOs) conserved across *Wolbachia*, an unusually high rate in contrast to 0% duplications that are observed in almost all other *Wolbachia* (supplementary fig. S4, Supplementary Material online). Second, a BlobPlot analysis of WOLB1015 showed the *Wolbachia* contigs spread over an unusually wide coverage range from 1 × to 1000 × , consistent with the presence of multiple *Wolbachia* in this assembly (supplementary fig. S9, Supplementary Material online). The WOLB1015 assembly had also failed to pass the QC criteria in the original study (Scholz et al. 2020). Therefore, instead of the mixed WOLB1015 assembly, the manually curated assembly of supergroup F *Wolbachia w*MoviF generated in this manuscript was used for all downstream analyses.

### Genome Sequence Comparisons between Various *Wolbachia*

**Fig. 2.**
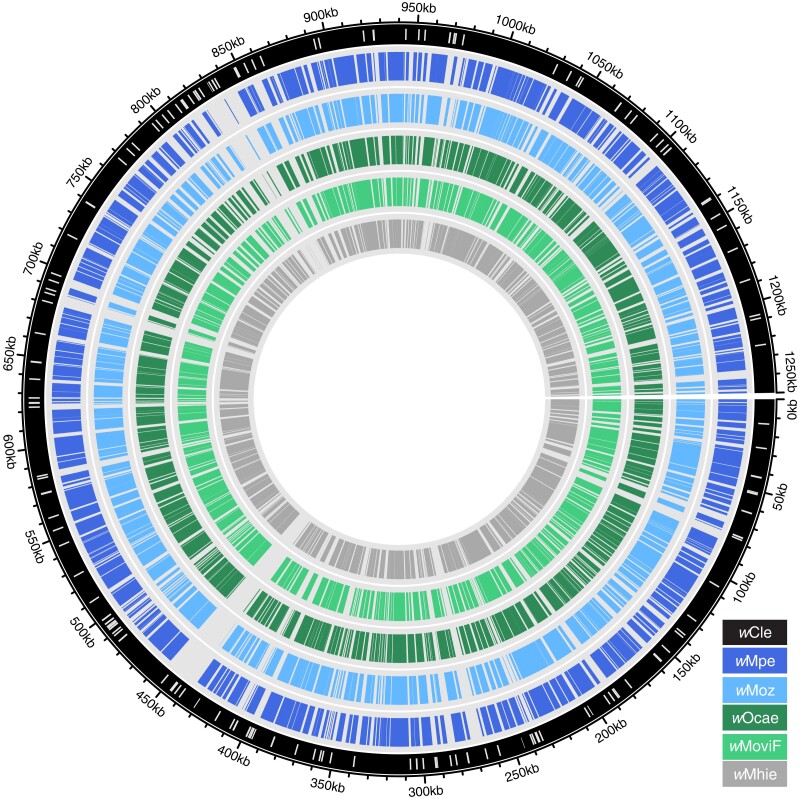
Gaps in the assembled *Wolbachia* genomes correspond mostly to IS elements. Whole genome alignments of *w*Mpe (blue ring), *w*Moz (light-blue ring), *w*Ocae (green ring), *w*MoviF (light green ring), and *w*Mhie (innermost gray ring) to the *w*Cle chromosome (outermost black circle) are visualized as a circos plot. The white bars mark the locations of the IS elements in the *w*Cle genome. The gaps in the alignments of various genomes to the *w*Cle genome overlap extensively with IS elements in the *w*Cle genome.

Analysis of whole genome alignments of the four newly assembled genomes to the complete *w*Cle genome showed that most of the regions absent from *w*Moz, *w*Mpe, *w*Ocae, or *w*Mhie assemblies corresponded to regions containing insertion sequence (IS) element transposons in the *w*Cle genome (fig. 2). This indicates that most of the protein coding regions have been recovered in these draft genome assemblies, and the missing regions largely correspond to the IS elements, which due to their repetitive nature present a technical challenge to assemble using only short-read data (Lefoulon et al. 2019; Sinha et al. 2019).

Comparisons of sequence similarity between all supergroup F *Wolbachia* genomes, and representatives from other supergroups including *Wolbachia* from filarial nematodes (Family Onchocercidae), a plant parasitic nematode *Pratylenchus penetrans* (Family Pratylenchidae), and arthropod hosts (supplementary table S1, Supplementary Material online) were performed using genome-wide average nucleotide identity (gANI, supplementary fig. S10*a*, Supplementary Material online) and digital DNA–DNA hybridization (dDDH) scores (supplementary fig. S10*b*–*d*, Supplementary Material online). The dDDH scores provide more sensitivity when comparing closely related species (Meier-Kolthoff et al. 2013). The *w*Mpe:*w*Moz pair was found to have a dDDH score of 71.7% (supplementary fig. S10*b*, Supplementary Material online). For comparison, the dDDH scores for other closely related sympatric *Wolbachia* are: 96.1% for *w*Oo:*w*Ov pair from *Onchocerca* spp. and 93.3% for *w*Bm:*w*Bpa pair from *Brugia* spp. (supplementary fig. S10*c*and*d*, Supplementary Material online). These scores suggest *w*Mpe and *w*Moz have diverged substantially from each other as compared to the *w*Oo:*w*Ov or *w*Bm:*w*Bpa pairs, most likely due to them having split from their common ancestor before the *w*Bm:*w*Bpa or *w*Oo:*w*Ov split.

### Orthology Analysis, Gene Function, and Pathway Annotations

Protein-coding gene sequences from NCBI-PGAP annotations were obtained for all 123 *Wolbachia* genomes available in GenBank (supplementary table S1, Supplementary Material online). For orthology analysis, protein-coding genes from the four new genomes, in addition to 77 genomes selected to represent all supergroups, were used (see column H, supplementary table S1, Supplementary Material online). The OrthoFinder analysis of a total 82,406 proteins encoded by these 81 genomes identified 1,751 orthogroups, 99 of which represent SCOs conserved across all the analyzed *Wolbachia* (supplementary table S2, Supplementary Material online). For the four new supergroup F *Wolbachia*, at least 96.9% of all predicted proteins could be assigned to an orthogroup (supplementary table S2, Supplementary Material online).

Functional annotations of the proteins encoded by the *w*Moz, *w*Mpe, *w*MoviF, and *w*Ocae genomes were obtained using the eggNOG database (supplementary table S3, Supplementary Material online). Additionally, biosynthetic pathway annotations for the *w*Cle genome were obtained from the KEGG database, and corresponding orthologs were identified in the *w*Moz, *w*Mpe, *w*MoviF, *w*Ocae, and *w*Mhie proteomes. Genes encoding members of various metabolic pathways characteristic of *Wolbachia* proteomes, namely the heme pathway, purine and pyrimidine biosynthesis pathways, riboflavin metabolism, and Type IV secretion systems, were found to be mostly present in *w*Moz and *w*Mpe (supplementary table S4, Supplementary Material online). Ribosomal protein subunits, a common target for anti-*Wolbachia* drugs such as doxycycline, as well as candidate drug targets such as pyruvate phosphate dikinase, PPDK (Raverdy et al. 2008), were also present in both *w*Moz and *w*Mpe.

#### Biotin Biosynthesis Genes Are Absent From All Supergroup F *Wolbachia* Except *w*Cle

The bedbug *Wolbachia w*Cle genome harbors an operon encoding the enzymes involved in biosynthesis of biotin, which has been acquired via LGT from another *Rickettsia* (Nikoh et al. 2014). The biotin produced by *w*Cle supports a nutritional mutualism between the bedbug and its *Wolbachia* (Nikoh et al. 2014). To investigate whether this feature is common to other supergroup F *Wolbachia*, a search for the corresponding orthologs was performed. No orthologs of any of the genes involved in the biotin biosynthesis pathway (genes *bioA*, *bioD*, *bioC*, *bioH*, *bioF*, and *bioB*) could be found in *w*Moz, *w*Mpe, *w*MoviF, or *w*Ocae proteomes. Additionally, when the sequencing reads from these four supergroup F *Wolbachia* were mapped against the *w*Cle genome, no alignments could be found within the region harboring the biotin operon, even though alignments could be obtained in the neighboring regions flanking the biotin operon (supplementary fig. S11, Supplementary Material online). The absence of biotin pathway genes is unlikely to be due to the incomplete nature of the draft assemblies. Given the high BUSCO score (at least 79%) of the assemblies, if the biotin pathway was genuinely present in the genomes, at least fragments of a few of the six genes in the biotin pathway would have been detected. In contrast, the seven genes for biosynthesis of riboflavin, another vitamin pathway important for the host–symbiont relationship (Li and Carlow 2012; Moriyama et al. 2015; Ju et al. 2020) were present in *w*Cle as well as all other supergroup F *Wolbachia* (supplementary table S4, Supplementary Material online). Thus, biotin supplementation might be unique to the bedbug and its *Wolbachia* association but is not the basis of mutualism between other supergroup F *Wolbachia* and their corresponding filarial or arthropod hosts.

### Prophage Remnants and Mobile Genetic Elements in Supergroup F *Wolbachia* Genomes

**Fig. 3.**
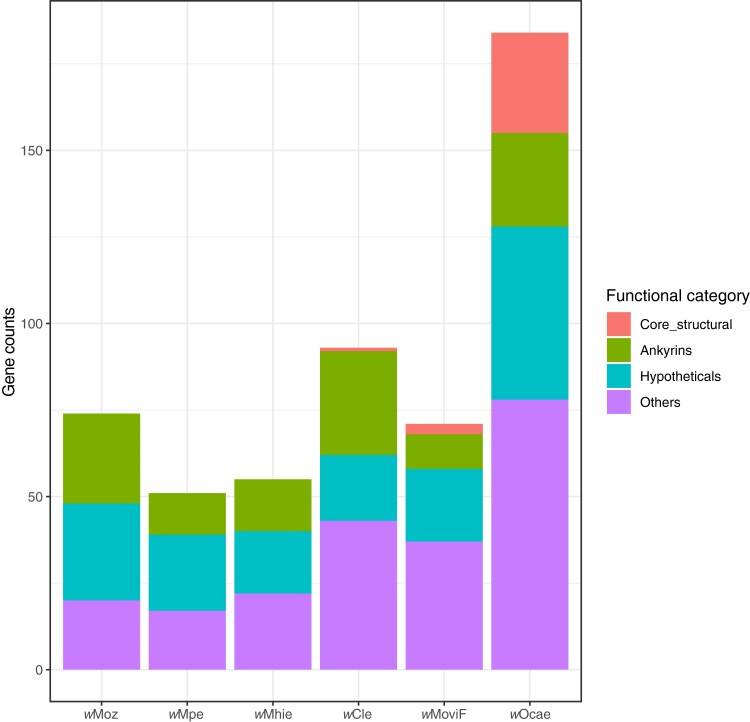
Numbers of phage-derived genes and their functional distributions in supergroup F *Wolbachia.* Phage-derived genes in each *Wolbachia* were categorized according to their predicted protein function. Phage-derived genes which had annotations other than “Hypothetical protein” but could not be classified as either “Core structural” or “Ankyrins” were included in the category “Others.” Only *w*Ocae has intact phage modules, whereas *w*MoviF and *w*Cle have only one and two core structural phage genes, respectively. Phage structural genes were absent from filarial *Wolbachia w*Moz, *w*Mpe, and *w*Mhie.

Many *Wolbachia* are naturally infected with lambda-like temperate phages, termed WO phages, which can integrate into *Wolbachia* genomes as prophages (Bordenstein and Bordenstein 2022). The phage-derived genes have been proposed to play important roles in generating genetic diversity, chromosomal evolution, and transfer of genes required for *Wolbachia* symbiosis (Bordenstein and Bordenstein 2022). Phage-derived genes in all supergroup F *Wolbachia* were identified via a comprehensive search for phage-derived orthogroups (fig. 3; supplementary table S5, Supplementary Material online) against a recently described database specific to *Wolbachia* phages (Bordenstein and Bordenstein 2022). In the *w*Moz and *w*Mpe genomes, 37 and 33 phage-derived orthogroups were found respectively, of which 32 were present in both *Wolbachia*. Among the gene members of these phage-derived orthogroups, “hypothetical protein” was the most common annotation (*n* = 22) followed by “ankyrin domain protein” (*n* = 12). The filarial *Wolbachia w*Mhie had 32 phage-associated orthogroups and shared 22 of these with *w*Moz and *w*Mpe. Neither *w*Moz, *w*Mpe, nor *w*Mhie was found to possess any core components of phage structural modules, like the filarial *Wolbachia* from other supergroups. Among arthropod *Wolbachia* of supergroup F, *w*Ocae had 29 phage structural genes, *w*MoviF had only three such genes, whereas *w*Cle had only one, suggesting different dynamics of phage evolution in these *Wolbachia*.

Group II introns are common, phage-derived mobile elements (Bordenstein and Bordenstein 2022) that can be found in high copy numbers in many *Wolbachia* genomes: for example, 53 copies are found in the *w*AlbB genome (Sinha et al. 2019). Within the supergroup F genomes, the *w*Ocae genome was found to encode only two copies of Group II introns. No such genes could be detected in the *w*MoviF, *w*Moz, or *w*Mpe genome assemblies. One pseudogene was detected in both *w*Moz and *w*Mpe. No intact gene or pseudogene for these elements could be found in the *w*Mhie or *w*Cle genomes, as reported earlier (Lefoulon, Clark, Guerrero, et al. 2020a).

The *Wolbachia cifA*-*cifB* genes, which encode cytoplasmic incompatibility factors that play a key role in the reproductive biology of their arthropod hosts, are also derived from phages (Lindsey et al. 2018; Bordenstein and Bordenstein 2022). In supergroup F, no *cifA* or *cifB* genes could be detected in the genomes of arthropod *Wolbachia w*Cle or *w*MoviF. In *w*Ocae, multiple pseudogenes or fragments of *cifA* and *cifB* genes missing either the 5-prime or 3-prime ends were identified (supplementary table S6, Supplementary Material online). Because all the gene fragments were observed to be located at the termini of the assembled scaffolds, they are most likely to be pseudogenes disrupted by IS element insertions that tend to fragment the assemblies (Sinha et al. 2019). Thus, *w*Ocae also seems to be devoid of any intact and functional *cifA*-*cifB* gene pair. No *cifA* or *cifB* orthologs could be detected in filarial *Wolbachia w*Moz, *w*Mpe, or *w*Mhie.

### Phylogenomic Analysis Encompassing All *Wolbachia* Genomes

**Fig. 4.**
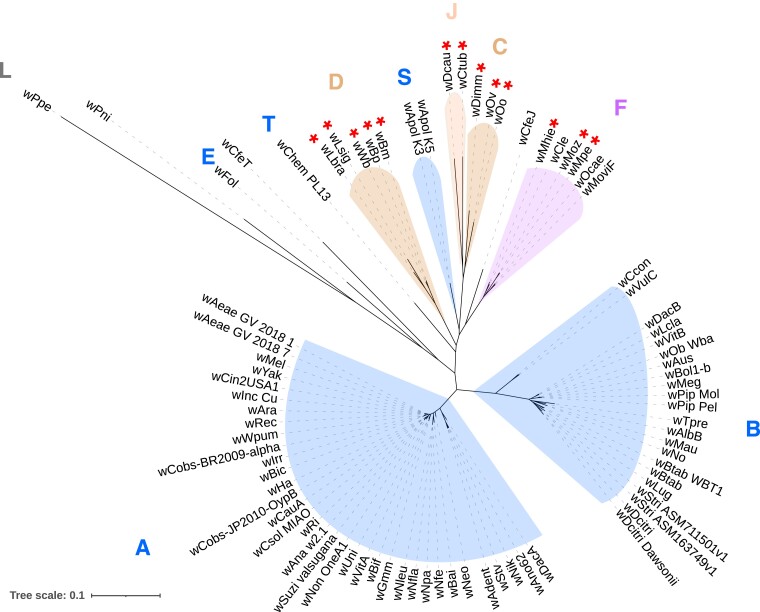
Global phylogenomic analysis of all *Wolbachia* genomes. Phylogenomic tree based on gene sequences of 91 SCOs conserved across 81 *Wolbachia* representing all supergroups. Supergroups comprised of *Wolbachia* exclusively from arthropod hosts are marked in blue, whereas the supergroups comprised exclusively of filarial *Wolbachia* are marked in orange. The supergroup F, which has members from both arthropod and filarial hosts, is marked in purple background. The red asterisks denote the *Wolbachia* with a pseudogenized or absent bacterioferritin gene.

To determine the evolutionary relationships between the supergroup F *Wolbachia* and other *Wolbachia*, as well as within supergroup F, comprehensive phylogenomic analyses were performed. Using a total of 127 *Wolbachia* genomes, including all 123 annotated genomes available from GenBank (supplementary table S1, Supplementary Material online) as input for OrthoFinder, 46 SCOs that are conserved across all available genomes were identified. The corresponding phylogenetic tree based on their multiple sequence alignment spanning 8,394 amino acids was produced (supplementary fig. S12, Supplementary Material online). For a further refinement of the tree, all incomplete or redundant genomes were excluded from analysis, except the genomes within supergroup F (see column H, supplementary table S1, Supplementary Material online). OrthoFinder analysis on these selected 81 genomes identified 99 SCOs (supplementary table S2, Supplementary Material online). Maximum likelihood trees with a partitioned model were built from the combined supermatrix of 62,160 nucleotides (fig. 4) and 20,470 amino acids (supplementary fig. S13, Supplementary Material online). The four new genomes were robustly placed within supergroup F in this analysis. In this tree, the *Wolbachia w*CfeJ from the cat flea *C. felis*, which has not been assigned any supergroup yet, was found to be closest to supergroup F.

**Fig. 5.**
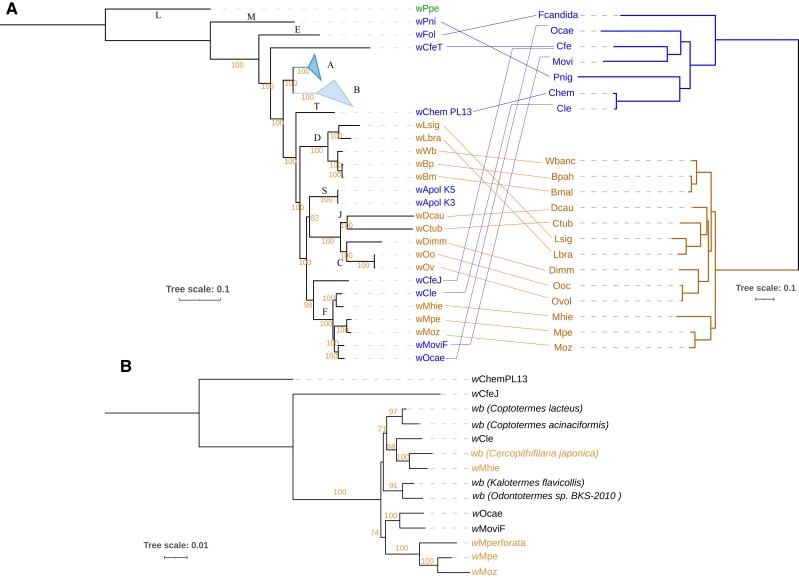
Two independent lineages of *Wolbachia*-filaria symbiosis within supergroup F. *A*) Mid-point rooted phylogenomic tree of *Wolbachia* based on gene sequences of 91 SCOs conserved across *Wolbachia* juxtaposed with a phylogenetic tree of various *Wolbachia* hosts based on 1,336 orthogroups. All filarial species, their *Wolbachia* and the lines depicting host-*Wolbachia* relationships are in orange. The arthropod hosts, their *Wolbachia* and their connecting lines are in blue. The bootstrap values are shown along the respective branches. Supergroups A and B comprise of 35 and 22 *Wolbachia* genomes respectively and are represented together as blue triangles. *Wolbachia* supergroups are denoted near the root node of each supergroup. *B*) Phylogenetic tree of supergroup F *Wolbachia* based on a supermatrix of 8 genes (*ftsZ*, *fbpA*, *coxA*, *gatB*, *hcpA*, *groEL*, *dnaA*, 16*S* rRNA), total sequence length 10,374 nucleotides, also supports at least two independent lineages of filarial *Wolbachia*. All *Wolbachia* from *Mansonella* hosts, including *M. perforata* are placed in a clade adjacent to the clade harboring arthropod *Wolbachia w*Ocae and *w*MoviF, whereas the *Wolbachia w*Mhie and *w*Cjaponica from filarial hosts *M. hiepei* and *C. japonica* are in a separate clade that is more closely related to the arthropod *Wolbachia w*Cle. Filarial *Wolbachia* are marked with orange font. Only the standard bootstrap values higher than 70 are shown.

Remarkably, the filarial *Wolbachia w*Moz and *w*Mpe were placed in a clade different from the clade harboring *w*Mhie, the other filarial *Wolbachia* in this supergroup (fig. 5*A*). The filarial *Wolbachia w*Moz and *w*Mpe were most closely related to the arthropod *Wolbachia w*MoviF and *w*Ocae, whereas the filarial *Wolbachia w*Mhie was more closely related to the arthropod *Wolbachia w*Cle. The topology of the *Wolbachia* phylogeny was also compared with the phylogeny of multiple *Wolbachia* hosts (fig. 5*A*), and limited congruence was found between the two phylogenies (fig. 5*A*). Particularly, within supergroup F, the clear separation of the filarial *Wolbachia* into two separate, well-supported clades, and the discordant phylogenetic topology of their corresponding hosts suggests at least two horizontal transfers of *Wolbachia* leading to these two clades.

To expand the analysis of supergroup F *Wolbachia* beyond those with whole genome sequences, a phylogenetic tree based on MLST loci (*ftsZ*, *fbpA*, coxA, gatB, hcpA) and *groEL*, *dnaA*, and 16S rRNA genes was constructed based on data available in GenBank (supplementary table S7, Supplementary Material online). This allowed including additional supergroup F *Wolbachia*, two from filarial hosts (*M. perforata* and *Cercopithifilaria japonica*) and four from different termite hosts. The corresponding sequences from *w*CfeJ, the *Wolbachia* found to be closest to supergroup F (fig. 5*A*), were used as an outgroup. A more distant *Wolbachia*, *w*ChemPL13 from supergroup T, which is a sister clade to supergroups F, C, J, S, and D was also included. This expanded tree (fig. 5*B*) also robustly supports the separation of the *Mansonella Wolbachia* clade from the *w*Mhie clade. Interestingly, the only other known filarial *Wolbachia* in supergroup F, from the deer tick nematode *C. japonica*, is placed in a clade with *w*Mhie, with the arthropod *Wolbachia w*Cle sharing a common ancestor with both these filarial *Wolbachia*. Together these observations demonstrate at least two distinct filarial *Wolbachia* lineages in supergroup F.

### Convergent Loss of Bacterioferritin across Filarial *Wolbachia*

Genome reduction and gene loss is a recurrent theme in the evolution of endosymbionts (Andersson and Kurland 1998), and the genomes of filarial *Wolbachia* are often found to be even more reduced as compared to their arthropod counterparts. A study of the genes lost during this evolutionary transition can therefore reveal genes that are either potentially dispensable or have been selected against during the evolution of filaria-*Wolbachia* symbiosis. The orthology relationships across 81 *Wolbachia* genomes (supplementary table S2, Supplementary Material online) were analyzed to look for orthogroups that were lost exclusively either in arthropod-associated or filaria-associated *Wolbachia*. Although no consistent orthogroup loss was found across arthropod *Wolbachia*, a striking loss of the bacterioferritin gene was observed in filarial *Wolbachia* across all supergroups C, D, J, and F (fig. 4). Within supergroup F, at least two independent losses of the bacterioferritin gene were observed, one in the *Mansonella Wolbachia* clade and the other in the *w*Mhie clade (fig. 5*A*). These pseudogenes had missing canonical start codons and premature stop codons resulting in truncated proteins about 75% of the length of the intact protein (115 aa in *w*Mpe, 125 aa in *w*Moz, 121 aa in *w*Mhie as compared to 160 aa in *w*Cle). The bacterioferritin proteins functions as a complex of 24 subunits forming a spherical cage to trap heme molecules (Rivera 2017) and a truncated protein is likely to disrupt this complex structure. Within supergroup F, all filarial *Wolbachia*, namely *w*Moz, *w*Mpe, and *w*Mhie, contain a pseudogenized bacterioferritin, even though their most closely related arthropod *Wolbachia* have the corresponding gene intact. Because the *w*Moz:*w*Mpe pair is placed in a separate clade from *w*Mhie (fig. 5), our results suggest that the *bfr* gene has been pseudogenized independently in the two separate lineages.

Interestingly, pseudogene fragments of the *bfr* gene in various stages of degradation could be found across all sequenced filarial *Wolbachia* (supplementary fig. S15*a*, Supplementary Material online). In supergroups C and J, although *bfr* pseudogenes were not found in the NCBI-PGAP annotations, blastx searches could identify leftover fragments of bacterioferritin in the corresponding genomic region (supplementary fig. S15*a*, Supplementary Material online). This genomic region was observed to be syntenic across all analyzed genomes (supplementary fig. S15*a*, Supplementary Material online), confirming that the lack of the *bfr* gene is not due to assembly artifacts.

Although the bacterioferritin locus in the RefSeq annotations of *Wolbachia w*Bpa from *B. pahangi* is annotated in NCBI as an intact CDS instead of a pseudogene, a closer look at its sequence and multiple sequence alignment with other bacterioferritin genes points to the loss of the canonical start codon ATG, similar to the corresponding sequence in *w*Bm, as well as other nucleotide insertions and deletions downstream (supplementary fig. S15*b*, Supplementary Material online), suggesting the pseudogenization of this gene in *w*Bpa as well. In *w*Bm, the *bfr* locus has a premature stop codon resulting in truncated protein with 138 amino acids instead of the complete 160 amino acids. The predicted peptide sequences corresponding to these pseudogenes showed multiple premature stop codons and frameshifts in both *w*Bm and *w*Bpa (supplementary fig. S15*c*, Supplementary Material online).

The dN/dS ratio, the ratio of nonsynonymous to synonymous substitutions for the *bfr* locus in *w*Bpa, was found to be higher than the corresponding locus in *w*Bm, (supplementary table S9, Supplementary Material online). Prior to dN/dS calculations, analysis of transitions and transversions with respect to genetic divergence plots and calculation of substitution saturation index (Xia et al. 2003) was performed to ensure that the bacterioferritin locus has not undergone substitution saturation across these *Wolbachia* (supplementary fig. S16, Supplementary Material online). Further, the dN/dS values for *bfr* locus in *w*Bm and *w*Bp were also compared against the distribution of genome-wide dN/dS ratios for all potential pseudogenes detected in these genomes using Pseudofinder (Syberg-Olsen et al. 2022). This analysis showed the dN/dS values for the *bfr* locus in *w*Bm and *w*Bpa are substantially higher than the median values of genome-wide dN/dS (supplementary fig. S17, Supplementary Material online). Together, these analyses confirm that bacterioferritin is undergoing pseudogenization in all filarial *Wolbachia*.

### Analysis of Pseudogenes Shared Within the Supergroup F *Wolbachia*

**Fig. 6.**
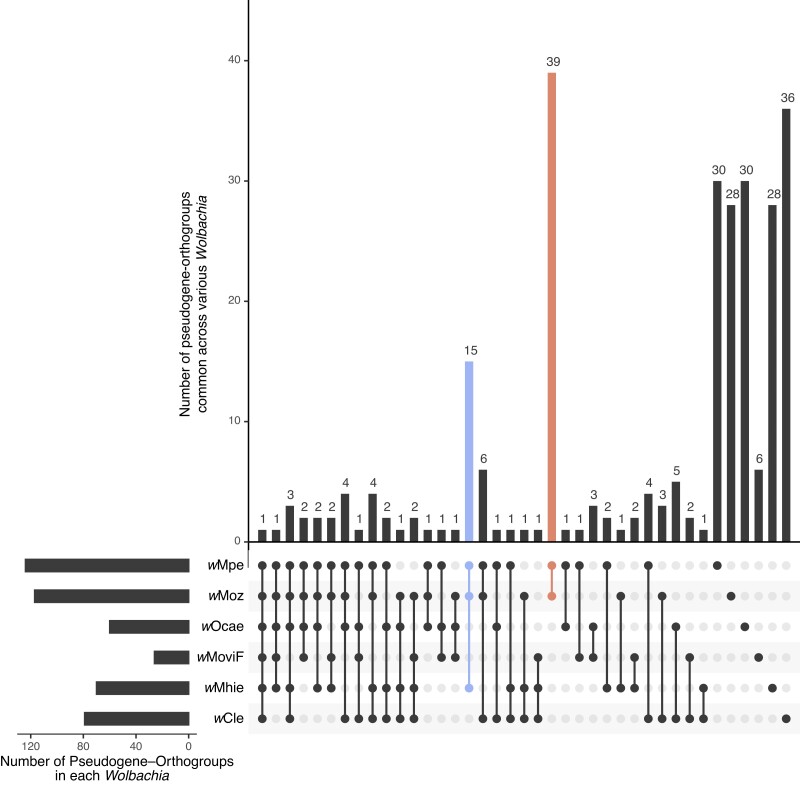
Pseudogenes shared between supergroup F *Wolbachia.* The orthogroups corresponding to the ancestral gene of each pseudogene were compared across different *Wolbachia*. Each *Wolbachia* harbors its unique set of pseudogenes, reflecting their origin as evolutionary accidents. The largest set of shared pseudogenes is observed between the closely related *w*Moz and *w*Mpe *Wolbachia* (*n* = 39, dark orange bar), suggesting either a shared common ancestor or convergent evolution within their respective *Mansonella* hosts. The next largest shared set of pseudogenes is found among the *Wolbachia w*Moz, *w*Mpe, and *w*Mhie (*n* = 15, blue bar), which are phylogenetically in different clades, indicating that the corresponding genes have undergone convergent loss of function across the three filarial *Wolbachia* in supergroup F. Transposons such as IS elements were excluded from this analysis due to their tendency to frequently convert into pseudogenes.

Intracellular bacterial symbionts, such as *Wolbachia*, are expected to accumulate pseudogenes due to genetic drift over evolutionary timescales (Andersson and Kurland 1998). However, the observation of two independent *bfr* pseudogenization events within supergroup F *Wolbachia* suggests that additional factors, such as convergent evolution through selection against certain genes could play a role in pseudogene formation. To explore the contributions of these factors, pseudogenes shared between various supergroup F *Wolbachia* were analyzed. This involved first determining the orthogroup corresponding to the ancestral gene of each pseudogene (supplementary table S8, Supplementary Material online). These “pseudogene-orthogroups” were then compared with identify those unique or shared across different *Wolbachia* (fig. 6). For each *Wolbachia*, the majority of its pseudogene-orthogroups were found to be unique to its genome, whereas typically very few (*n* = 1 to 6) were shared between different *Wolbachia*, consistent with the genetic drift model. An exception to this trend was the high number (*n* = 15) of genes that were found to be pseudogenized in each of the three filarial *Wolbachia w*Moz, *w*Mpe, and *w*Mhie, whereas their closest sister arthropod *Wolbachia* had intact versions of the corresponding genes (fig. 6). The bacterioferritin pseudogene described above is one of these 15 genes. Because *w*Moz and *w*Mpe are placed in a clade separate from *w*Mhie, these 15 pseudogenes have most likely arisen from independent, convergent loss in filarial *Wolbachia* across different lineages within supergroup F. The highest number of shared pseudogene-orthogroups was observed in the *w*Moz:*w*Mpe pair (*n* = 39). The corresponding pseudogenes might have arisen either in the last common ancestor of *w*Moz and *w*Mpe or through convergent evolution specific to *Wolbachia* within *Mansonella* or a combination of both factors.

## Discussion

*Wolbachia* endosymbionts play critical roles in the biology of their arthropod and filarial hosts, and there is great interest in understanding their diverse functions through genomic approaches. The enigmatic supergroup F in *Wolbachia* phylogeny is the only supergroup comprising symbionts of arthropod as well as filarial nematode hosts. In this report, four new *Wolbachia* genomes from supergroup F are described, expanding the number of genomes from two to six in this underrepresented supergroup. Two genomes are from *w*Moz and *w*Mpe, endosymbionts of Mansonella spp., important filarial parasites of humans, whereas the other two genomes are from *w*Ocae and *w*MoviF, endosymbionts of the mason bee *O. caerulescens* and the sheep ked *M. ovinus* respectively.

Because *Wolbachia* are intracellular symbionts that cannot be cultured independently of their hosts, it is not possible to obtain their DNA independent of the host and the associated microbiome, presenting a challenge for accurate genome assembly. In the case of *w*Moz and *w*Mpe, complex human clinical samples were used as the source material, containing a mixture of DNA from human blood, the filarial parasite host, its intracellular symbiont *Wolbachia*, as well as other constituents of the human microbiome. Similarly, for *w*Ocae and *w*MoviF genomes, the publicly available sequencing data were derived from field-collected, wild isolates of their arthropod hosts, and comprised DNA from the arthropod host, *Wolbachia*, as well as the arthropod microbiome. Therefore, a metagenomic assembly and binning pipeline, customized to each of the datasets, were necessary and played a critical role in obtaining high quality *Wolbachia* genomes.

The BlobTools-based binning approach separates the assembled scaffolds based on their GC content and relative read coverage, which also provides a means for an accurate determination of *Wolbachia* titers via the measurement of relative copy numbers of genomes of *Wolbachia* versus its host. This principle has been used in a metagenomic study for *Wolbachia* titer calculations across multiple hosts (Scholz et al. 2020), and for highlighting sex-specific differences in *Wolbachia* levels in *D. immitis* (Kumar et al. 2013). Given the longstanding controversy around the detection of *Wolbachia* in *M. perstans* (Grobusch et al. 2003; Keiser et al. 2008; Coulibaly et al. 2009; Gehringer et al. 2014; Debrah et al. 2019; Sandri et al. 2021), determination of *Wolbachia* titers in *Mansonella* is particularly important and has implications for antibiotic use as a treatment (Gehringer et al. 2014). In the current study, the titer in the isolate Mpe1 was found to be 30 *Wolbachia* cells per microfilaria. In the isolate Mpe2, although the presence of *Wolbachia* sequences could be detected, their depth and breadth of coverage was too low for estimating the titer reliably. Importantly, this low *Wolbachia* coverage in Mpe2 is not simply due to an overall low sequencing depth, as the corresponding genome for the host *M. perstans* could be successfully assembled at a high coverage that was comparable to the isolate Mpe1. As both Mpe1 and Mpe2 samples were collected in Cameroon, these observations demonstrate that even within the same geographical region, *Wolbachia* levels can widely vary between different isolates. For the *M. ozzardi* isolate Moz1, *Wolbachia* was detected but could not be assembled due to overall low sequencing depth obtained for this sample. In isolate Moz2, the titer was determined to be 37 *Wolbachia* cells per microfilaria, similar to Mpe1, and to levels reported from a laboratory strain of *B. malayi* (McGarry et al. 2004). The presence of *Wolbachia* in all *Mansonella* isolates described here, albeit at varying levels, and in another human parasite *Mansonella* spp. “DEUX” (Sandri et al. 2021), together with the successful clearance of *M. perstans* microfilariae with doxycycline (Coulibaly et al. 2009; Debrah et al. 2019), supports the use of antibiotics as a promising treatment for mansonellosis.

The *Wolbachia* from supergroup F present a unique opportunity to investigate the evolutionary history of closely related arthropod and filarial *Wolbachia* because all other supergroups are comprised exclusively of either arthropod or filaria associated *Wolbachia*. A global phylogenomic analysis of 127 *Wolbachia* genomes, including all supergroup F genomes, confirmed the placement of the 4 new genomes (*w*Moz, *w*Mpe, *w*MoviF, and *w*Ocae) in supergroup F and identifies *w*CfeJ as the most closely related and robust outgroup to this supergroup. The phylogenomic analyses revealed two distinct clades of filarial *Wolbachia* within supergroup F. The *w*Moz and *w*Mpe pair shared a common ancestor with the arthropod *Wolbachia w*Ocae and *w*MoviF, whereas *w*Mhie shared a different common ancestor with the bed bug *Wolbachia w*Cle, indicating multiple events of horizontal transfer of *Wolbachia* between arthropod and filarial hosts. While this scenario has previously been proposed based on a few gene loci (Casiraghi et al. 2005; Lefoulon et al. 2012, 2016), the genome-wide phylogeny presented here, based on a supermatrix of 91 genes, with 2 new filarial and 2 new arthropod *Wolbachia* genomes, provides the most comprehensive and robust evidence in support of multiple horizontal transfers across the two host phyla. In addition to horizontal transfers across hosts, the widespread occurrence and transmission of *Wolbachia* infections across different animal phyla can also occur through cladogenic inheritance or introgression. The relative contributions of these three transmission modes have been analyzed for *Wolbachia* in closely related host systems, such as in the *Nasonia* species complex (Raychoudhury et al. 2009) and different *Drosophila* species complex (Turelli et al. 2018; Cooper et al. 2019). Horizontal transfer of *Wolbachia* has been confirmed as the mechanism explaining very similar *Wolbachia* observed in the reproductively isolated *Drosophila* species *D. simulans* and *D. ananassae* (Turelli et al. 2018). The widely diverged arthropod and filarial hosts in supergroup F are also reproductively isolated species and phyla that harbor very similar *Wolbachia.* Therefore, horizontal transfer of *Wolbachia* between the two phyla is the most likely explanation for their observed phylogeny. This conclusion is further supported by the incongruence between the phylogenies of supergroup F *Wolbachia* and their hosts, where *w*Mhie and *wb C. japonica* are sister *Wolbachia* branching off the same node (fig. 5*B*), but their hosts are from different clades, ONC5 and ONC4, respectively (Lefoulon et al. 2016). Therefore, the *w*Mpe/*w*Moz and the *w*Mhie/*wb C. japonica* lineages are distinct and unlikely to be vertically inherited from a single filarial ancestor with an ancestral *Wolbachia*.

An interleaved pattern of clades with arthropod- or filarial-*Wolbachia* members is also evident beyond supergroup F, across the global phylogenomic analysis encompassing all available *Wolbachia.* The *Wolbachia* supergroups C and J with filarial hosts are a sister clade to the arthropod-only supergroup S. Deeper in the phylogeny, the filarial supergroup D shares a common ancestor with supergroups C, J, S, F, and *w*CfeJ. Furthermore, the supergroup T *Wolbachia w*ChemPL13 from a bed bug shares a common ancestor with the supergroup D, as well as the clade containing S, J, C, F, and *w*CfeJ. These phylogenetic patterns and the incongruence between the host-*Wolbachia* cophylogenies indicate multiple horizontal transfers between the arthropod and filarial hosts, but the direction of these transfers remains challenging to infer from the phylogenomic data alone.

The striking observation of a convergent pseudogenization of the bacterioferritin gene in filarial *Wolbachia* enables formulation of hypotheses regarding direction of *Wolbachia* transfer within supergroup F. At least two independent losses of the bacterioferritin gene were observed in this supergroup, one in the *w*Moz-*w*Mpe clade and the other in the *w*Mhie clade, whereas their sister *Wolbachia* from arthropod hosts had an intact *bfr* gene. This suggests that an intact *bfr* gene is likely the ancestral state, as a reversion from a pseudogene to an intact gene is very unlikely. This intact ancestral *bfr* gene could have been present in an ancestral *Wolbachia* that was associated with either a nematode or arthropod host. Because all the extant filarial *Wolbachia* with available genomes lack the *bfr* gene, the likelihood of a filarial nematode as an ancestral host is relatively low. This hypothesis is based on a limited number of filarial *Wolbachia* genomes, which harbor the *bfr* gene at various stages of pseudogenization (supplementary fig. S15*a*, Supplementary Material online). If future sampling of more diverse filarial *Wolbachia* identifies any such *Wolbachia* with intact bacterioferritin, the current hypothesis will no longer be applicable.

The loss of bacterioferritin has important implications for the obligate relationship between *Wolbachia* and filarial hosts. Bacterioferritin is known to be a critical regulator of heme homeostasis in bacteria, serving as a store for heme that can be used during heme deficiency, or sequestering excess heme to prevent toxicity (Carrondo 2003). In arthropods, the presence of *Wolbachia* provides a fitness benefit by increasing fecundity during conditions of iron deficiency (Brownlie et al. 2009), while during iron excess, *Wolbachia* protect their insect hosts from iron induced toxicity by upregulating bacterioferritin (Kremer et al. 2009). Heme is an essential nutrient required by filarial worms, but they lack a heme biosynthetic pathway. Heme provisioning by *Wolbachia* has been proposed to play a key role in the obligate host-symbiont relationship (Foster et al. 2005; Gill et al. 2014). The lack of bacterioferritin in filarial *Wolbachia* suggests that *Wolbachia* cells can produce but no longer store heme intracellularly, making it available for uptake by the host filaria, and in doing so, affording a fitness advantage for the parasite. Under this model, the various ancestral filarial *Wolbachia* might have had an intact bacterioferritin gene to begin with, but in the lineages that lost this gene due to genetic drift, the freeing up of heme for the filarial host would have provided a strong enough fitness advantage to drive the *bfr*-lacking *Wolbachia* to fixation in filarial hosts and initiating an obligate relationship. Although genome reduction and gene loss, usually via genetic drift, is a recurrent theme in the evolution of endosymbionts (Andersson and Kurland 1998), the loss of bacterioferritin gene across multiple filarial *Wolbachia* is a unique example of convergent gene loss accompanying the adaptation to a new ecological niche. Further research into the role of bacterioferritin and heme metabolism can better elucidate their role in different host-*Wolbachia* systems.In summary, this study provides four newly assembled supergroup F *Wolbachia* genomes. A global phylogenomic analysis including all sequenced *Wolbachia* genomes together with these new genomes has provided strong support for multiple lineages of filarial *Wolbachia* in supergroup F, as well as uncovered an intriguing loss of bacterioferritin from all filarial *Wolbachia*. Further studies of these four new genomes are expected to shed more light on the evolution and nature of symbiosis, as well as the identification of new treatments for mansonellosis.

## Materials and Methods

### Ethics Statement

All research involving human subjects was approved by the appropriate committees and performed in accordance with all relevant guidelines and regulations. Informed consent was obtained from all participants or their legal guardians.

For *M. perstans*, ethical clearance was obtained from the National Institutional Review board, Yaoundé (REF: *N*° 2015/09/639/CE/CNERSH/SP), and administrative clearance from the Delegation of Public Health, South-West region of Cameroon (Re: R11/MINSANTE/SWR/RDPH/PS/259/382). Approval for the study was granted by the “National Ethics Committee of Research for Human Health” in Cameroon. Special consideration was taken to minimize the health risks to which any participant in this study was exposed. The objectives of the study were explained to the consenting donor after which they signed an informed consent form. The participant's documents were assigned a code to protect the privacy of the study subject. At the end of the study, the donor received a cure of mebendazole (100 mg twice daily for 30 days).

For *M. ozzardi*, study protocols were approved by the Institutional Review Board of the Institute of Biomedical Sciences, University of São Paulo, Brazil (1133/CEP, 2013). Written informed consent was obtained from all patients or their parents or guardians if participants were minors aged <18 years. Diagnosed infections were treated with a single dose of 0.2 mg/kg of ivermectin after blood sampling (De Almeida Basano et al. 2018; Lima et al. 2018, 39).

### Parasite Materials and DNA Extraction

*M. ozzardi* DNA was prepared from microfilariae collected from blood samples of individuals who participated in a previous study in Brazil (Lima et al. 2018). A sample containing a high microfilaria load was selected for genome sequencing and is denoted as Moz1 in the present study. A Venezuelan isolate of *M. ozzardi* microfilariae was generously donated by Izaskun Petralanda in 1,989 and is denoted as Moz2 in this study. Genomic DNA was prepared from Moz2 microfilariae by Proteinase K digestion followed by phenol/chloroform extraction and ethanol precipitation as well as drop dialysis (https://www.neb.com/protocols/2013/09/16/drop-dialysis) and then stored at –20 °C. Two independent isolates of *M*. *perstans* microfilariae, denoted as Mpe1 and Mpe2 in this study, were collected on nylon filters from blood samples from Cameroon (Poole et al. 2019). Mpe1 DNA was extracted using a Genomic DNA Tissue MicroPrep kit (Zymo Research, USA) as described previously (Poole et al. 2019). DNA from Mpe2 was isolated as described above for Moz2. DNA quantity was determined using a Qubit dsDNA HS Assay kit in conjunction with a Qubit 2.0 Fluorometer as directed by the manufacturer (Life Technologies, USA).

### Illumina Library Construction and Sequencing

Prior to library construction, the NEBNext Microbiome DNA enrichment kit (New England Biolabs Inc., USA) was used to enrich parasite DNA and reduce human DNA contamination (except for the Moz2 sample). The enrichment process is based on selective binding and removal of CpG-methylated DNA islands present in human genome but absent from nematode and *Wolbachia* genomes. The relative amounts of DNA from nematode and *Wolbachia* therefore remains unaffected after the enrichment step. The preparation of Illumina libraries from Mpe1 and Moz1 samples has been described as part of a previous study (Poole et al. 2019), and a similar protocol was used for Mpe2 and Moz2, using the NEBNext Ultra II FS DNA Library Prep Kit (New England Biolabs Inc., USA) as described by the manufacturer. Following PCR amplification with different index primers to enable multiplexing, the libraries were size selected using NEBNext Sample Purification Beads (NEB cat. # E7767) following manufacturer's instructions. The approximate insert size and concentration of each library was determined using a 2100 Bioanalyzer with a high sensitivity DNA chip (Agilent Technologies, USA). Two Mpe2 libraries with insert sizes of approximately 500 and 800 bps and two Moz2 libraries with insert sizes of approximately 500 and 950 bps were constructed. Libraries were diluted to 4 nM with 10 mM Tris and 0.1 mM EDTA pH 8 for sequencing. Due to the A:T rich nature of filarial genomes, Phi X DNA was added to balance base pair composition, then sequenced using the Illumina MiSeq and NextSeq500 platforms (paired end, 150 bps).

### Metagenomic Assembly and Binning

Raw reads were processed using the BBTools package v38.51 (https://sourceforge.net/projects/bbmap/). Duplicate raw reads and bad tiles were removed using the clumpify.sh and filterbytile.sh utilities. Adapter trimming as well as removal of phiX reads and reads with 13-nt or longer stretches of the same nucleotide (poly-G, -C, -A, or-T) were performed using bbduk.sh. Human host reads were removed by mapping against the human genome (grch38) using the bbmap.sh utility. The quality metrics of the processed reads at each step were assessed using FastqC v0.11.9 (https://www.bioinformatics.babraham.ac.uk/projects/fastqc/).

Reads from different runs of the libraries prepared from the same genomic DNA sample were combined and used as an input for the assembly of the metagenome using metaSpades v3.14.0 (Nurk et al. 2017). Input reads were mapped back to the assembled scaffolds using bowtie2 v.2.4.1 (Langmead and Salzberg 2012). Binning of metagenomic scaffolds into metagenome assembled genomes of *Mansonella* and *Wolbachia* was performed using the BlobTools v1.1.1 software (Laetsch and Blaxter 2017) and additional manual curation. The bins annotated as “Proteobacteria” and “Nematoda” were analyzed further to retrieve sequences genuinely originating from *Wolbachia*. A cluster of “Proteobacteria” scaffolds in the blobplots of each of the metagenomes was analyzed using blastn against the *w*Cle genome to verify their *Wolbachia* origin. These scaffolds were subsequently collected as respective *Wolbachia* assemblies from each of the isolates. The combined size of scaffolds collected from the Moz1 isolate was only 120 kb, much smaller than a 1 Mb genome typically seen for *Wolbachia*, and so were not analyzed further. Similarly, in the Mpe2 sample, only 12 scaffolds with a total size of 15 kb were identified as candidate *Wolbachia* sequences and were not analyzed further. The tight clusters of “Proteobacteria” scaffolds in the blobplots of Moz2 and Mpe1 were classified as the *w*Moz and *w*Mpe genome assemblies respectively. For the remaining clusters of metagenome scaffolds that were also marked “Proteobacteria” by BlobTools but had a distinctly different %GC as compared to the *Wolbachia* scaffolds, blastn searches against the NCBI-nt database were performed to verify that they did not originate from the *Wolbachia* genome. All the bioinformatics programs were run using the default parameters unless otherwise stated.

The genome sequence of *Wolbachia w*Ocae from the mason bee *O. caerulescens* was assembled from Illumina reads available under NCBI SRA accession SRR1221705, originally described in a previous study of arthropod *Wolbachia* (Gerth et al. 2014). The reads were assembled into metagenomic scaffolds using metaSpades. A BlobTools analysis of the resulting assembly was used to identify the cluster of *Wolbachia* scaffolds.

The genome sequence of *Wolbachia w*MoviF from the sheep ked *M. ovinus* was assembled from raw Illumina reads corresponding to the NCBI SRA accession number ERR969522, which originated from a study of sheep ked endosymbionts (Nováková et al. 2015) that were also used recently for *Wolbachia* genome mining (Scholz et al. 2020). In the first round of assembly using all reads as input to metaSpades assembler, potential *Wolbachia* scaffolds were identified using BlobTools. However, a preliminary blastn analysis of these scaffolds indicated a contaminating genome from supergroup A or B *Wolbachia*, in addition to the expected supergroup F *Wolbachia* in this sample. Due to the complicated nature of this dataset, additional iterations of metagenomic assembly and binning were performed using only the reads that mapped to a “bait” database containing all known *Wolbachia* genomes. The mapping of reads to bait databases was performed using bowtie2 (v.2.4.1). The bait database was updated to include the newly assembled sequences, and a new subset of reads was obtained by mapping to this updated database. These reads were then used as input for another round of metagenomic assembly. Candidate *Wolbachia* scaffolds were selected using BlobTools. Whole genome alignments of the assembled scaffolds to three representative and complete reference genomes, namely *w*Cle (supergroup F), *w*Mel (supergroup A), and *w*AlbB (supergroup B) were performed using nucmer v4.0.0beta2 (Marçais et al. 2018, 4) with the parameter “mincluster” set to 100.

### Genome Annotation and Comparative Analysis

For both *w*Mpe and *w*Moz genomes, as well as the *w*Ocae and *w*MoviF genomes, protein-coding genes, rRNA, tRNA, ncRNA genes, and pseudogenes were identified using the NCBI prokaryotic genome annotation pipeline (Tatusova et al. 2016). The completeness of the protein-coding content of all four genomes was assessed using the BUSCO pipeline v5.1.3 using the “proteobacteria_odb10” reference dataset (Simão et al. 2015). Syntenic blocks between various pairs of genomes were analyzed and visualized using the JupiterPlot tool (https://github.com/JustinChu/JupiterPlot), which uses minimap2 (v 2.17-r941) for whole-genome alignments (Li 2018). The parameters used in Jupiter plots are maxGap = 100,000; minBundleSize = 1,000; m_ref_contig_cutoff = 500; and gScaff = 500. The parameter “ng” was set to 100%, so that all the scaffolds from the two assemblies being compared are displayed in the Jupiter plots. Whole-genome alignments of *w*Moz, *w*Mpe, *w*Ocae, and *w*MoviF, as well as *w*Mhie, against *w*Cle as the reference genome were performed using the nucmer utility in MUMmer4 with default parameters. The resulting alignment blocks were visualized as concentric Circos plots (Krzywinski et al. 2009) drawn using the R package circlize v0.4.10 (Gu et al. 2014). For global sequence comparisons across multiple *Wolbachia* genomes, the average nucleotide identity (ANI) scores were calculated using the OrthoANIu tool v1.2 (Yoon et al. 2017). The pairwise ANI scores were used for hierarchical clustering of different *Wolbachia*, and a correlation plot was generated using R package corrplot v0.84 (https://github.com/taiyun/corrplot). For a more sensitive measure of sequence similarity and divergence between closely related *Wolbachia*, dDDH scores (Meier-Kolthoff et al. 2013) were computed using the recommended “formula 2” at the “Genome-to-Genome Distance Calculator” (GGDC) web-service (https://ggdc.dsmz.de/ggdc.php). The orthologs of protein coding genes across multiple *Wolbachia* genomes were inferred using OrthoFinder v2.4 (Emms and Kelly 2019).

IS elements were identified via the ISsaga web server (Varani et al. 2011) and by parsing the annotations in GenBank format from NCBI-PGAP pipeline. Other transposons and Group II introns were also inferred via parsing the GenBank files. Functional annotation of protein-coding genes was carried out using the eggNOG-Mapper (Huerta-Cepas et al. 2017) web server (http://eggnogdb.embl.de/#/app/emapper). The analysis of different metabolic pathways was based on the annotations of *w*Cle and *w*Bm genomes available in the KEGG database (Kanehisa et al. 2017).

### Phylogenomic Analysis

Phylogenomic analysis was carried out on three distinct sets of input genomes. The first set comprised 127 genomes and included 123 annotated *Wolbachia* genomes available in the NCBI GenBank database (supplementary table S1, Supplementary Material online) as well as the 4 genomes reported in this study. For a more robust and refined tree, a second set was used comprising 81 genomes selected from the 127 genomes, excluding incomplete genomes (except those within supergroup F) as well as redundant genomes, for example multiple isolates of the same *Wolbachia* (supplementary table S1, Supplementary Material online). The third set included only the genomes from supergroup F *Wolbachia*, and *w*CfeJ *Wolbachia* from the cat flea *Ctenocephalides felis* as an outgroup.

For each input set, OrthoFinder was used to identify SCOs conserved across all genomes within the set. Protein sequences corresponding to each orthogroup were aligned using mafft v7.149b (Nakamura et al. 2018). The multiple sequence alignments were trimmed using Gblocks v0.91b (Talavera and Castresana 2007). The trimmed blocks from each conserved orthogroup were then concatenated into a supermatrix, and the length of each alignment block was recorded in a nexus format partition file. The corresponding gene sequence tree was generated by collecting the transcript sequences corresponding to the SCOs conserved across all input *Wolbachia*. The DNA sequences corresponding to each conserved orthogroup were first aligned using mafft and then concatenated into a supermatrix sequence, with the lengths of the alignment blocks recorded in a nexus format partition file. Maximum likelihood trees were generated based on protein sequences as well as gene sequences, using the corresponding concatenated supermatrix sequence and partition files (Chernomor et al. 2016) as inputs to iqtree v2.1.2 (Nguyen et al. 2015). Automatic selection of substitution models was performed using ModelFinder (Kalyaanamoorthy et al. 2017) implementation in iqTree (iqTree command line option “-m MFP’). Evaluation of branch supports was performed using the iqTree implementations of 1) the SH-like approximate likelihood ratio test (Guindon et al. 2010) and 2) ultrafast bootstrap (Hoang et al. 2018) with 1,000 replicates each (iqTree command-line options “-B 1000 -alrt 1000 -abayes -lbp 1000”). The consensus trees were visualized using the iTOL webserver (Letunic and Bork 2021) and further annotated in Adobe Illustrator.

For a phylogenetic analysis of all supergroup F *Wolbachia* based on a combined supermatrix of multiple genes, the sequences for their MLST loci *ftsZ*, *fbpA*, *coxA*, *gatB*, *hcpA*, and other loci (*groEL*, *dnaA*, ftsZ and 16S rRNA) were obtained from their genome assemblies and GenBank. The corresponding sequences from *w*CfeJ was used as outgroup. Only the *Wolbachia* with sequences available for at least three of these eight loci were included in this analysis. The multiple sequence alignment of the combined supermatrix of sequences was performed using MUSCLE (Edgar 2004). Phylogenetic tree with automatic model selection, standard bootstrap calculations, and single branch tests was generated using iqtree (command-line options -b 1000 -alrt 1000 -abayes).

### Assembly and Annotation of *Wolbachia* Host Genomes and Transcriptomes for Phylogenomic Analysis

The protein coding genes of different *Wolbachia* hosts were obtained as follows. The genomes and proteomes for filarial nematodes *M. perstans*, *M. ozzardi* were recently described (Sinha et al. 2023). Curated proteomes from publicly available data for the filarial hosts *B. malayi*, *B. pahangi*, *Wuchereria bancrofti*, *Dirofilaria immitis*, *On. ochengi*, *On. volvulus*, *Litomosoides sigmodontis*, *L. brasiliensis*, *Dipetalonema caudispina*, and *Cruorifilaria tuberocauda* were the same as recently (Sinha et al. 2023). The genome sequence of *O. caerulescens* was obtained from the metagenomic assembly and blobplot analysis for *w*Ocae described above. Annotated genes for *C. lectularius* and *Folsomia candida* were obtained from NCBI assemblies GCF_000648675.2 and GCF_002217175.1 respectively. The *Pentalonia nigronervosa* genome was obtained from NCBI accession GCA_014851325.1. Modeling and masking of genomic repeats in *O. caerulescens* and *P. nigronervosa* genomes was performed using RepeatModeler version 2.0.3 (Flynn et al. 2020) and Dfam database (Storer et al. 2021). These repeats were soft-masked in the respective genomes using RepeatMasker 4.1.2-p1 (http://repeatmasker.org/). Gene predictions were carried out on WebAUGUSTUS online server () with the soft-masked genome as input. The soft-masked genomes obtained were used as input to gene prediction using online AUGUSTS web server (Hoff and Stanke 2013). The transcriptomes for *M. ovinus* and *C. hemipterus* were assembled de novo from SRA datasets SRR17267914 (Zhang et al. 2023) and SRR19217017 respectively. The reads were corrected using rcorrector (Song and Florea 2015) and assembled using Trinity-v2.8.5 (Haas et al. 2013). The longest super-transcripts were selected using transdecoder v5.5.0 (https://github.com/TransDecoder/TransDecoder), and arthropod sequences were selected using kraken version 2.0.9-beta (Wood and Salzberg 2014) and kraken-tools script extract_kraken_reads.py (Lu et al. 2022). Conserved orthologs and species tree for these nematode and arthropod hosts were obtained from OrthoFinder (Emms and Kelly 2019).

### Gene Loss and Pseudogene Analysis

OrthoFinder results, from the set of 81 genomes described above, were analyzed to identify gene loss events across different *Wolbachia*, by cataloging orthogroups which had gene members present in all arthropod *Wolbachia* but absent in filarial *Wolbachia*.

Pseudogenization of protein-coding genes is a major factor contributing to gene loss. Pseudogenes common across different supergroup F *Wolbachia* were identified as follows. A catalog of pseudogenes for each *Wolbachia* was first obtained from its NCBI-PGAP annotation. Next, for each pseudogene, the GenBank accession for its most likely intact ancestral protein was also obtained from the NCBI-PGAP annotations. For a phylogenetically consistent and robust analysis, instead of comparing these protein accessions directly, the corresponding ancestral orthogroup was retrieved from the results of OrthoFinder, and these “pseudogene-orthogroups” were compared across different *Wolbachia*. If the same orthogroup was found to be pseudogenized in more than one *Wolbachia*, the corresponding pseudogenes were designated as shared or common pseudogenes. The overlap between the ancestral pseudogene-orthogroups across different *Wolbachia* was visualized using the R package UpsetR (Conway et al. 2017). Pseudogenes derived from transposons and IS elements were excluded from this analysis.

The dN/dS values for *w*Bpa-bfr and *w*Mhie-*bfr* loci were calculated from pairwise alignments with intact genes from closely related *Wolbachia*, using PAL2NAL utility (Suyama et al. 2006). The substitution saturation index of both these loci was evaluated using Xia's test (Xia et al. 2003) using DAMBE (Xia 2018). The proportion of invariant sites at *bfr* locus for this analysis was obtained via PhyML (Guindon et al. 2010) analysis of 13 intact bacterioferritin genes from all supergroups. A global analysis of dN/dS values for all genes and pseudogenes for *w*Bpa and *w*Bma against the *w*ChemPL13 reference genome were obtained using Pseudofinder (Syberg-Olsen et al. 2022).

### Annotation of Phage-Derived Elements and Cytoplasmic Incompatibility Factors

Phage-derived genes and pseudogenes were annotated following the latest taxonomic classification scheme of *Wolbachia* phages (Bordenstein and Bordenstein 2022). The sequences of *Wolbachia* phage genes and pseudogenes were obtained based on the Phage-WO maps recently described (Bordenstein and Bordenstein 2022). Their best sequence matches in the proteomes of the set of 81 *Wolbachia* genomes were first identified using blastp and blastx searches. The orthogroups corresponding to these best hits were considered as phage-derived orthogroups, and the corresponding orthologs in supergroup F *Wolbachia* were annotated as phage-derived genes. Genes encoding IS-elements were excluded from this analysis to avoid mismatches and overcounting arising from the repeated and fragmented nature of genes in these families. Pseudogenes were annotated as phage-derived if their predicted ancestral proteins had an ortholog in the phage WO database. For *cifA* and *cifB* gene annotations, reference gene sequences were compiled from published data (Martinez et al. 2021) and used in blast searches against the proteomes from the 81 *Wolbachia* genomes used in the phylogenomic analysis above, and the corresponding orthogroups and orthologs within supergroup F *Wolbachia* were annotated as *cifA* and *cifB*.

## Supplementary Material

evad073_Supplementary_Data

## Data Availability

Raw Illumina reads for the *M. perstans* isolate Mpe1 and the *M. ozzardi* isolate Moz2 have been deposited in the NCBI SRA database under BioProject accession numbers PRJNA666671 and PRJNA666672, respectively. The assembled genome and PGAP annotations for the *Wolbachia w*Mpe and *w*Moz are available from the NCBI GenBank database with accession numbers JACZHU000000000 and JADAKK000000000, respectively. The annotated genomes for *w*Ocae and *w*MoviF are available as GenBank accessions JAHDJT000000000 and JAHRBA000000000, respectively, deposited under BioProject accessions PRJNA244005 and PRJNA739984, respectively.
