# Supplementary material for: Multiple Lineages of Nematode-Wolbachia Symbiosis in Supergroup F and Convergent Loss of Bacterioferritin in Filarial Wolbachia
Source: Genome Biol Evol. 2023 May 8;15(5):evad073. doi: 10.1093/gbe/evad073 (PMC10195089; doi:10.1093/gbe/evad073)

Figure Number	Title
S1	BlobTools based binning of the metagenomes assembled from *Mansonella ozzardi* isolates<br>(a) *M. ozzardi* isolate Moz1, from Brazil (b) *M. ozzardi* isolate Moz2, from Venezuela
S2	BlobTools based binning of the metagenomes assembled from *Mansonella perstan*s isolates<br>(a) *M. perstans* isolate Mpe1, from Cameroon (b) *M. perstans* isolate Mpe2, from Cameroon
S3	Jupiter plots show regions of conservation and synteny between (a) *w*Moz and (b) *w*Mpe in comparison to the *w*Cle genome
S4	BUSCO scores of various *Wolbachia*
S5	(a) Taxonomic binning of metagenomic scaffolds to identify *Wolbachia* from the mason bee *Osmia caerulescens* (b) Jupiter plot representation of whole-genome alignment between the Wolbachia wOcae assembly versus the complete reference genome of *w*Cle.
S6	BlobPlot for the assembly WOLB0012 shows a mixture of *Wolbachia* and host arthropod contigs
S7	Multiple proteobacterial endosymbionts are present in *Melophagus ovinus*
S8	*Melophagus ovinus* has a supergroup F *Wolbachia* as well as a supergroup A *Wolbachia*, requiring a customized binning pipeline (a - h).
S9	BlobPlot for the assembly WOLB1015 shows *Wolbachia* contigs spread over an unusually wide range of read-coverage, from 1X to 1,000X, indicating the presence of multiple *Wolbachia* in this assembly.
S10	Global comparisons between multiple *Wolbachia* genomes using (a) genome-wide Average Nucleotide Identity (gANI) scores, and digital DNA:DNA Hybridization (dDDH) scores between *Wolbachia* in (b) supergroup F (c) supergroup C (d) supergroup D
S11	No evidence for biotin biosynthesis genes in any supergroup F *Wolbachia* except *w*Cle
S12	Phylogenomic tree of 127 *Wolbachia* genomes based on protein sequences of 46 Single Copy Orthologs conserved across all *Wolbachia*
S13	Phylogenomic tree of 81 *Wolbachia* genomes based on protein sequences of 99 Single Copy Orthologs conserved across all *Wolbachia*.
S14	Convergent loss of bacterioferritin gene across all available filarial *Wolbachia* genomes (a) Protein alignments (b) Gene alignments (c) Syntenic genomic regions flanking the *bfr* gene in various *Wolbachia*.
S15	The bfr gene locus has not undergone substitution saturation across *Wolbachia*.
S16	dN/dS values for bacterioferritin locus in *w*Bm and *w*Bpa as compared to genome-wide dN/dS values for all pseudogenes in these *Wolbachia.*

Fig. S1. BlobTools based binning of metagenomes assembled from *Mansonella ozzardi* isolates (a) Moz1 and (b) Moz2

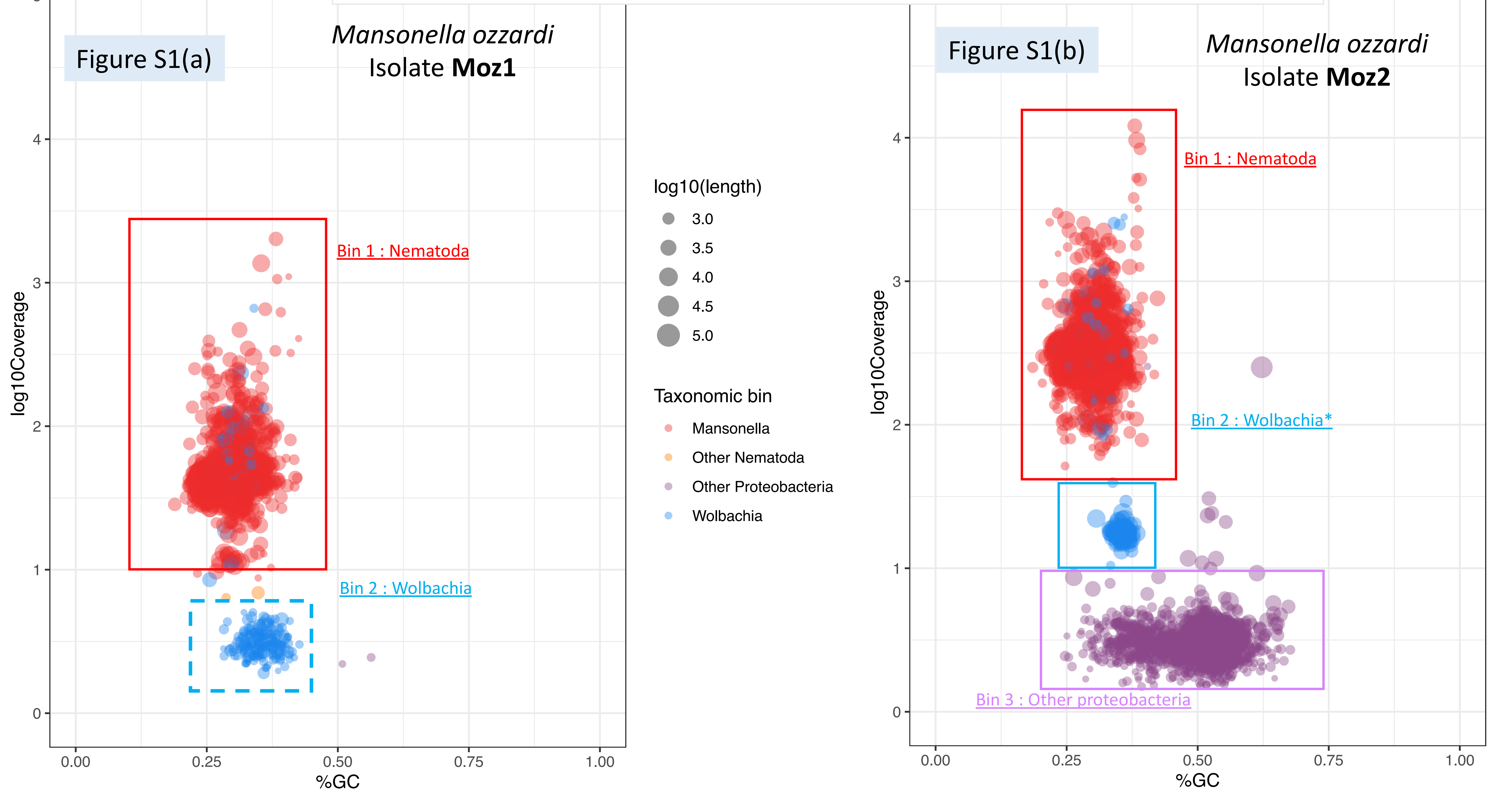

Fig. S2. BlobTools based binning of metagenomes assembled from *Mansonella perstans* isolates (a) Mpe1 and (b) Mpe2

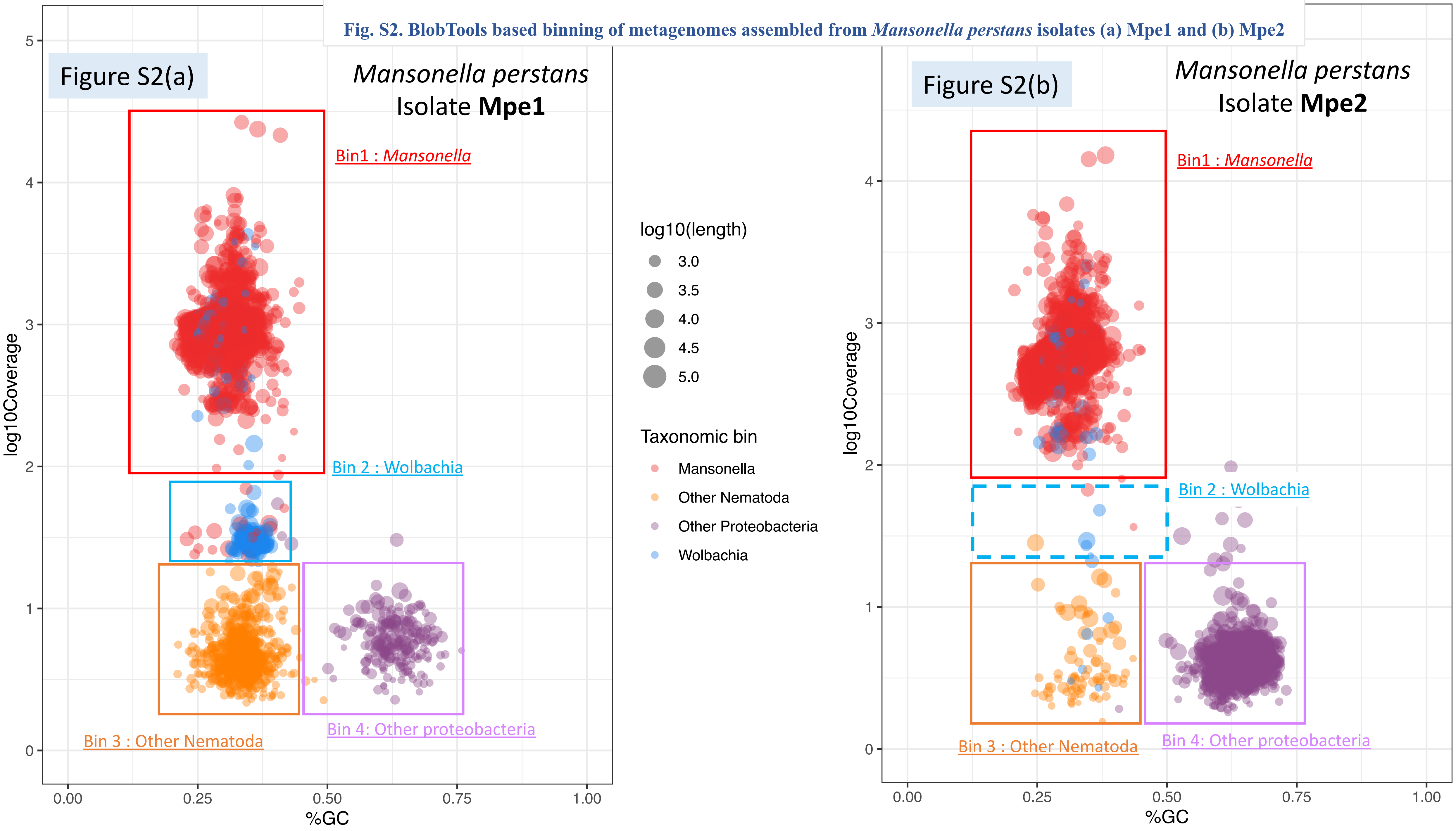

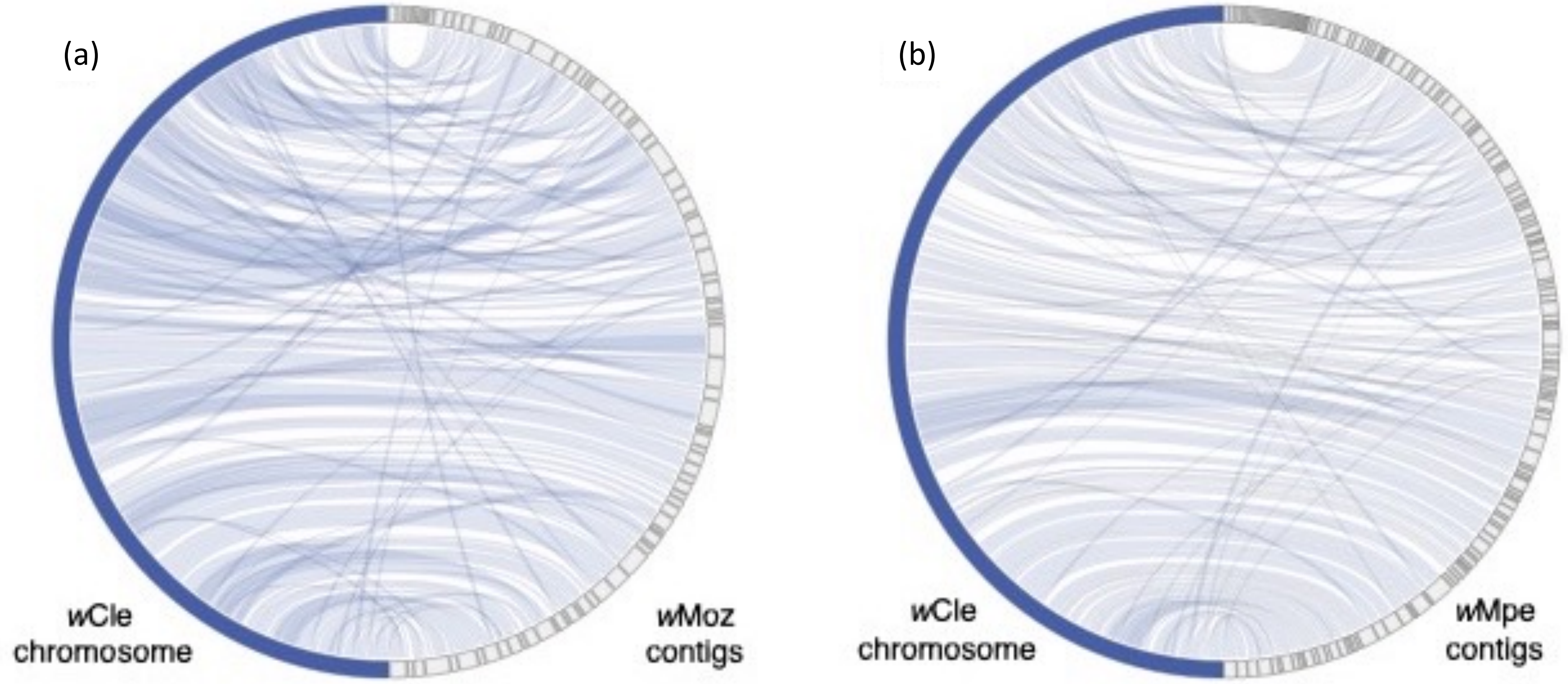

Figure S3. Jupiter plots show regions of conservation and synteny between (a) *w*Moz and (b) *w*Mpe in comparison to the *w*Cle genome

The *w*Cle chromosome is represented in linear form as the blue semi-circle on the left in both panels. The gray boxes represent contigs of (a) *w*Moz draft genome, and (b) *w*Mpe draft genome. The horizontal bands connect the conserved regions across the two *Wolbachia*. Regions of re-arrangements are shown as bands crisscrossing the horizontal bands of syntenic regions.

Figure S4. BUSCO scores of various Wolbachia

The BUSCO pipeline was used to measure the proportion of highly conserved, single copy orthologs (BUSCO groups). The set of reference BUSCO groups was set to the lineage "proteobacteria_odb10", which contains 219 BUSCOs derived from proteobacterial species. The *w*Oo genome has the lowest BUSCO score, marked by a vertical dotted line. The supergroups are indicated within a parenthesis after the corresponding *Wolbachia* names. The BUSCO scores of the *Wolbachia* genomes reported in this study are comparable to other *Wolbachia* genomes, indicating high quality of these draft genomes.

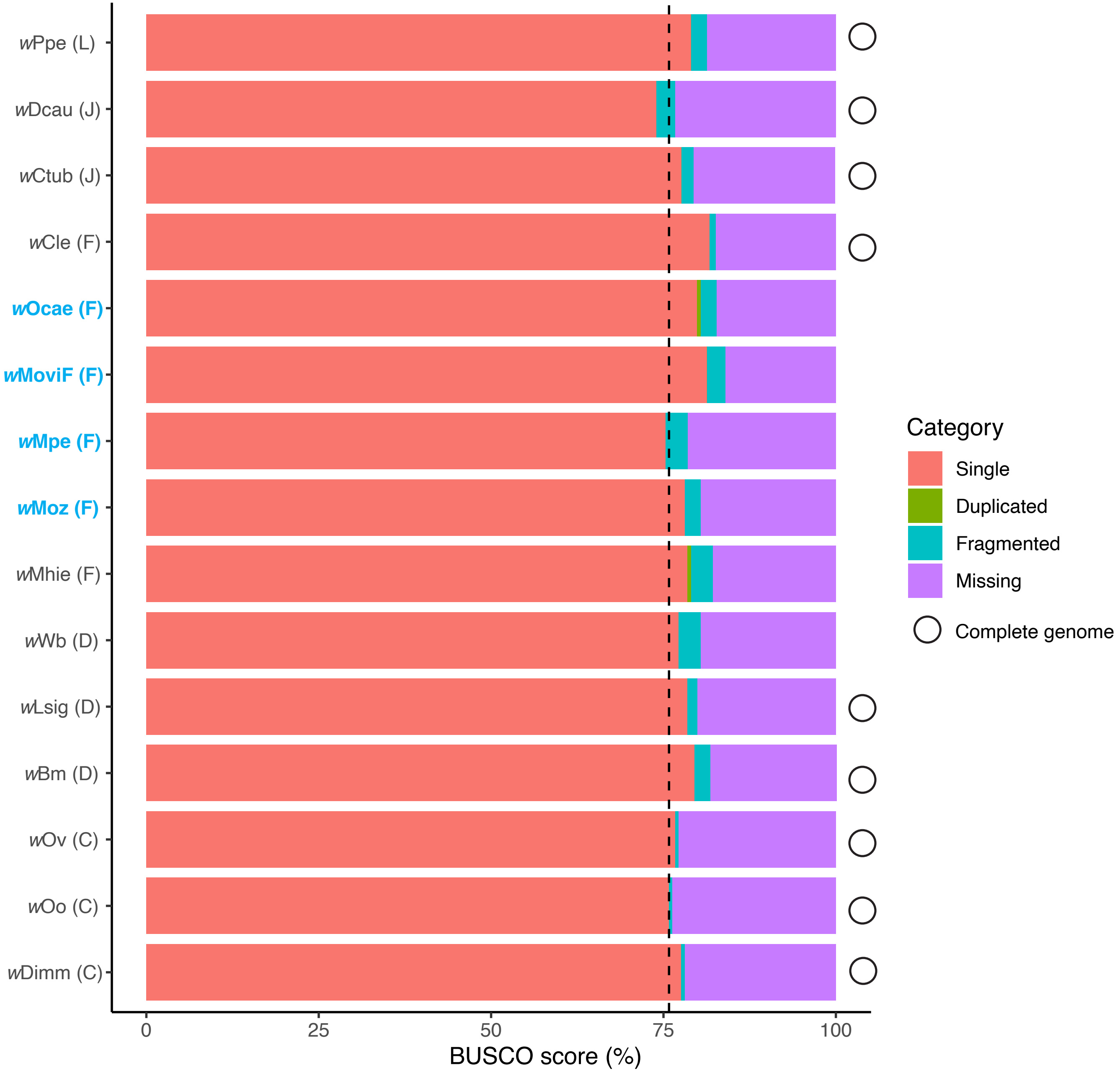

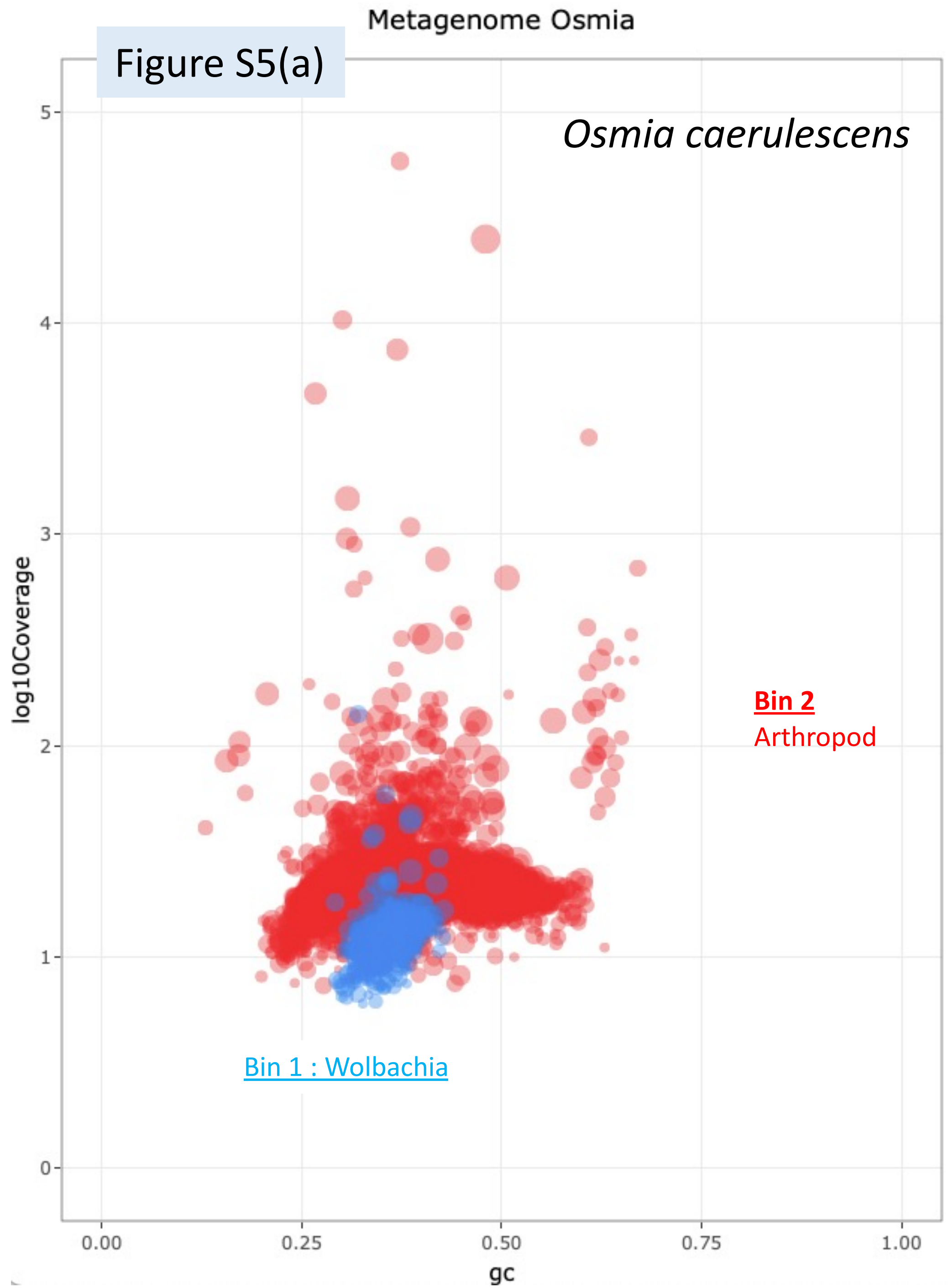

Figure S5. Taxonomic binning of metagenomic scaffolds to identify *Wolbachia* from the mason bee *Osmia caerulescens*

The cluster of scaffolds identified as *Wolbachia* by the BlobTools analysis are tightly clustered and well separated from the scaffolds from the arthropod host.

Figure S5(b)

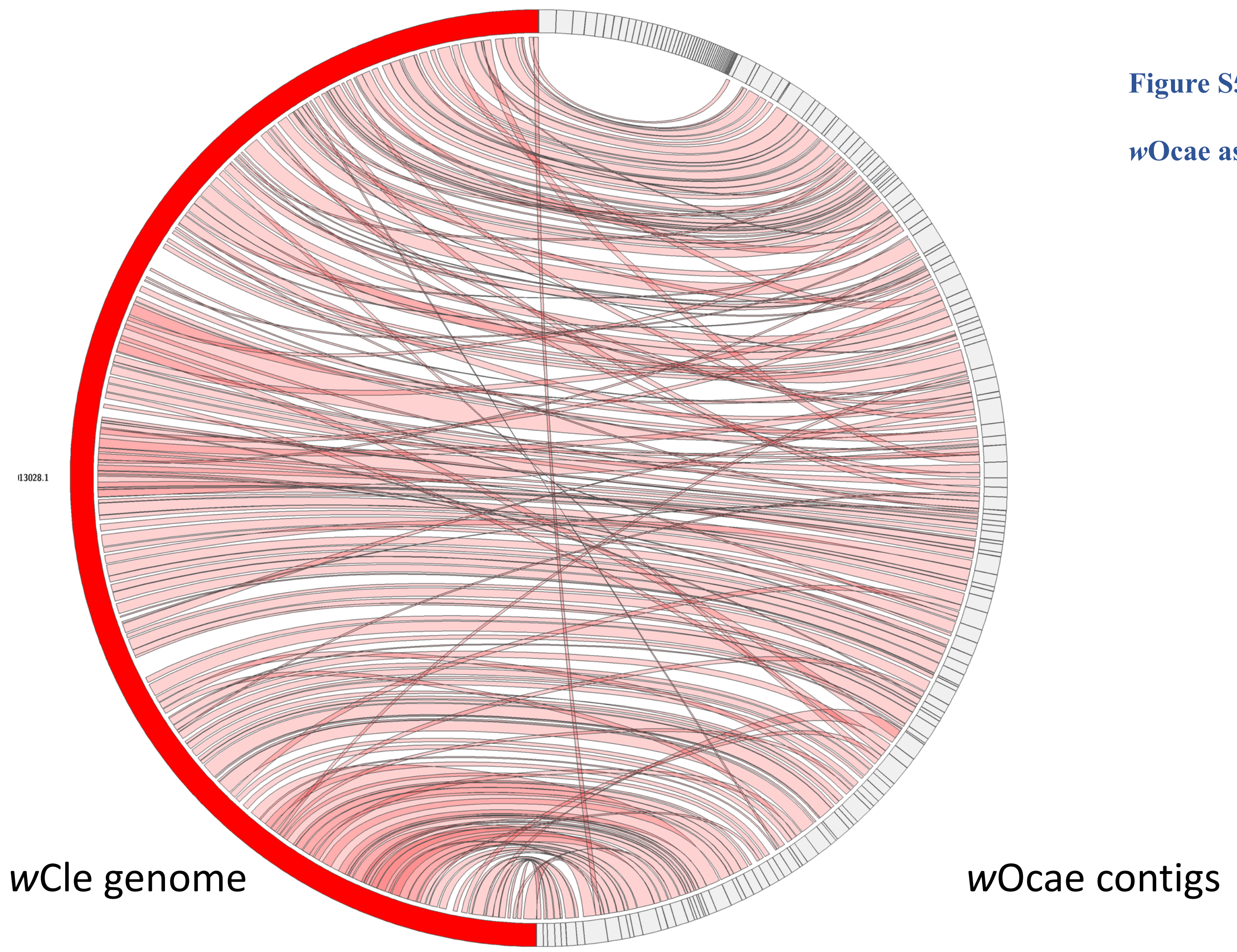

Figure S5. Jupiter plot representation of whole-genome alignment between the *Wolbachia* *w*Ocae assembly versus the complete reference genome of *w*Cle

Figure S6. BlobPlot for assembly WOLB0012 shows a mixture of *Wolbachia* and host arthropod contigs

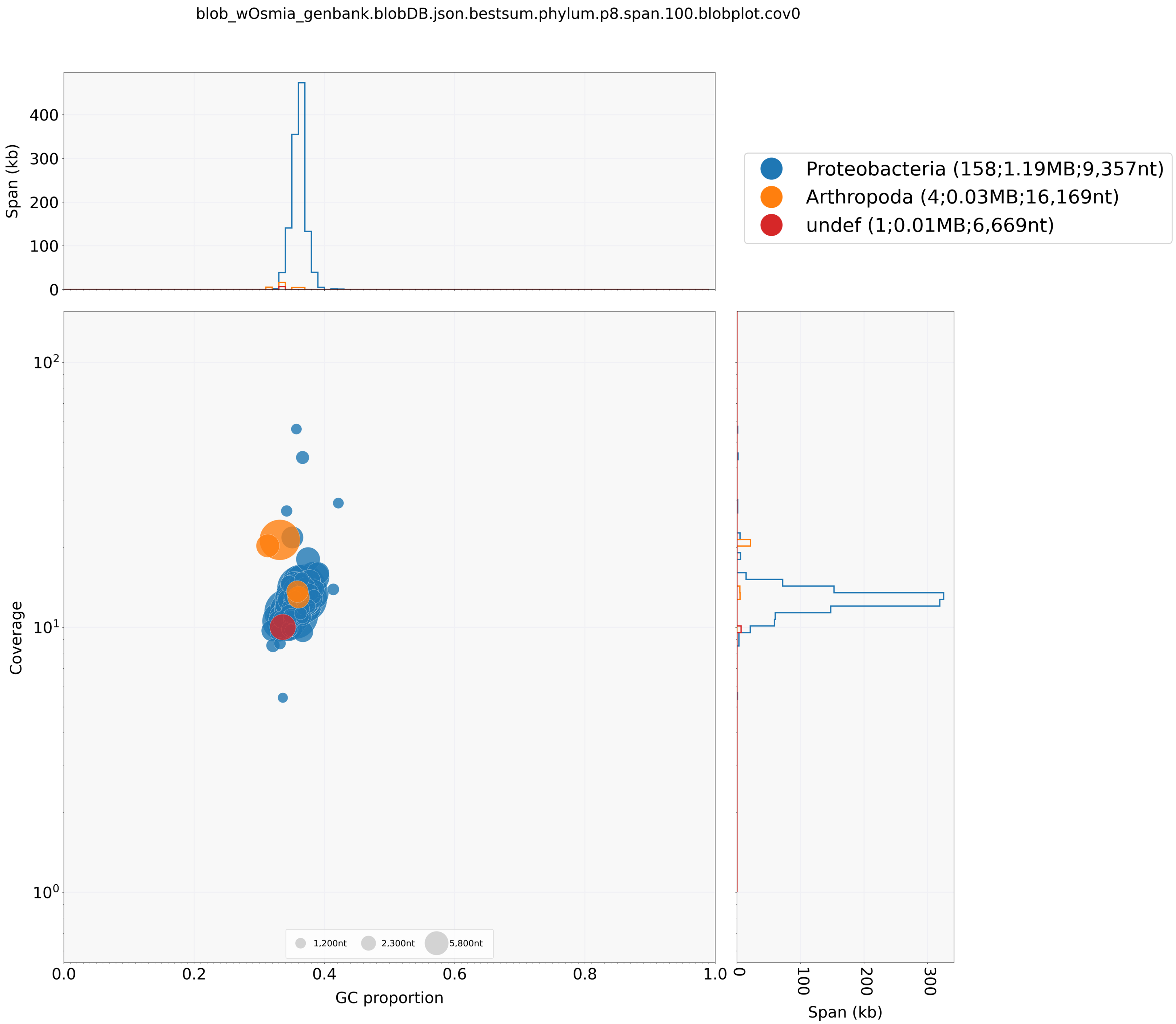

Fig. S7. Multiple endosymbionts in the complex microbiome of *Melophagus ovinus*

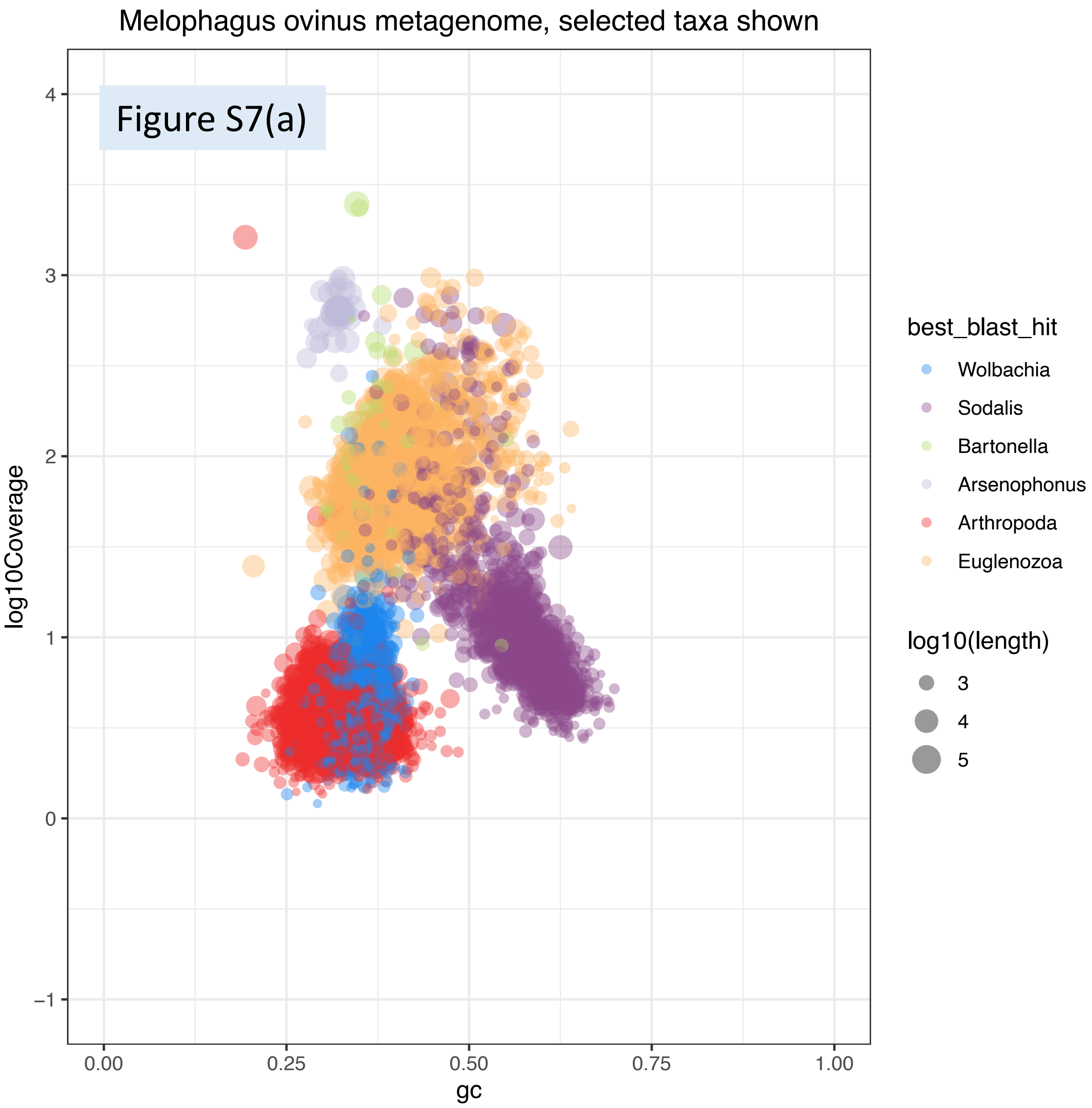

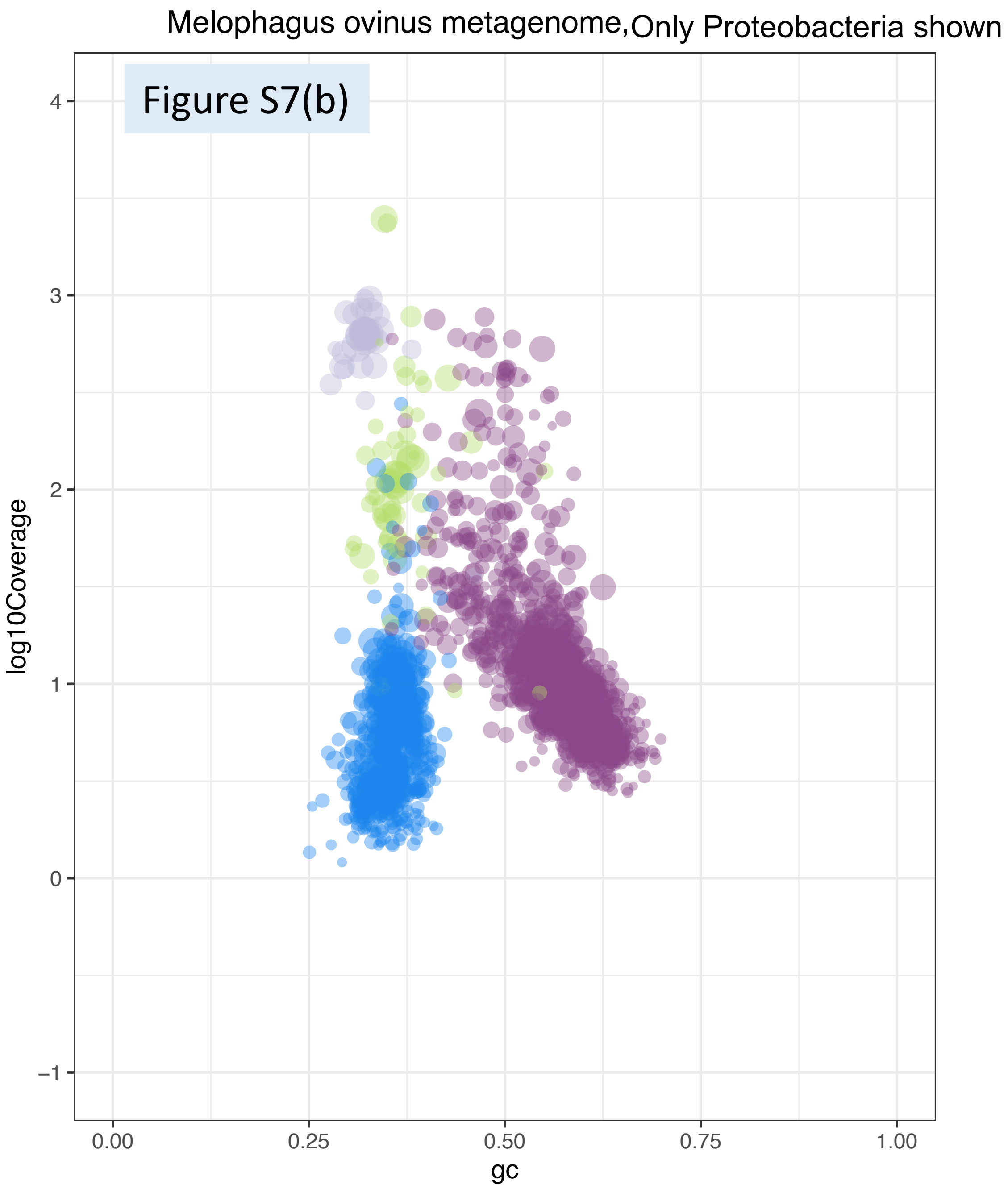

Figure S8(a)

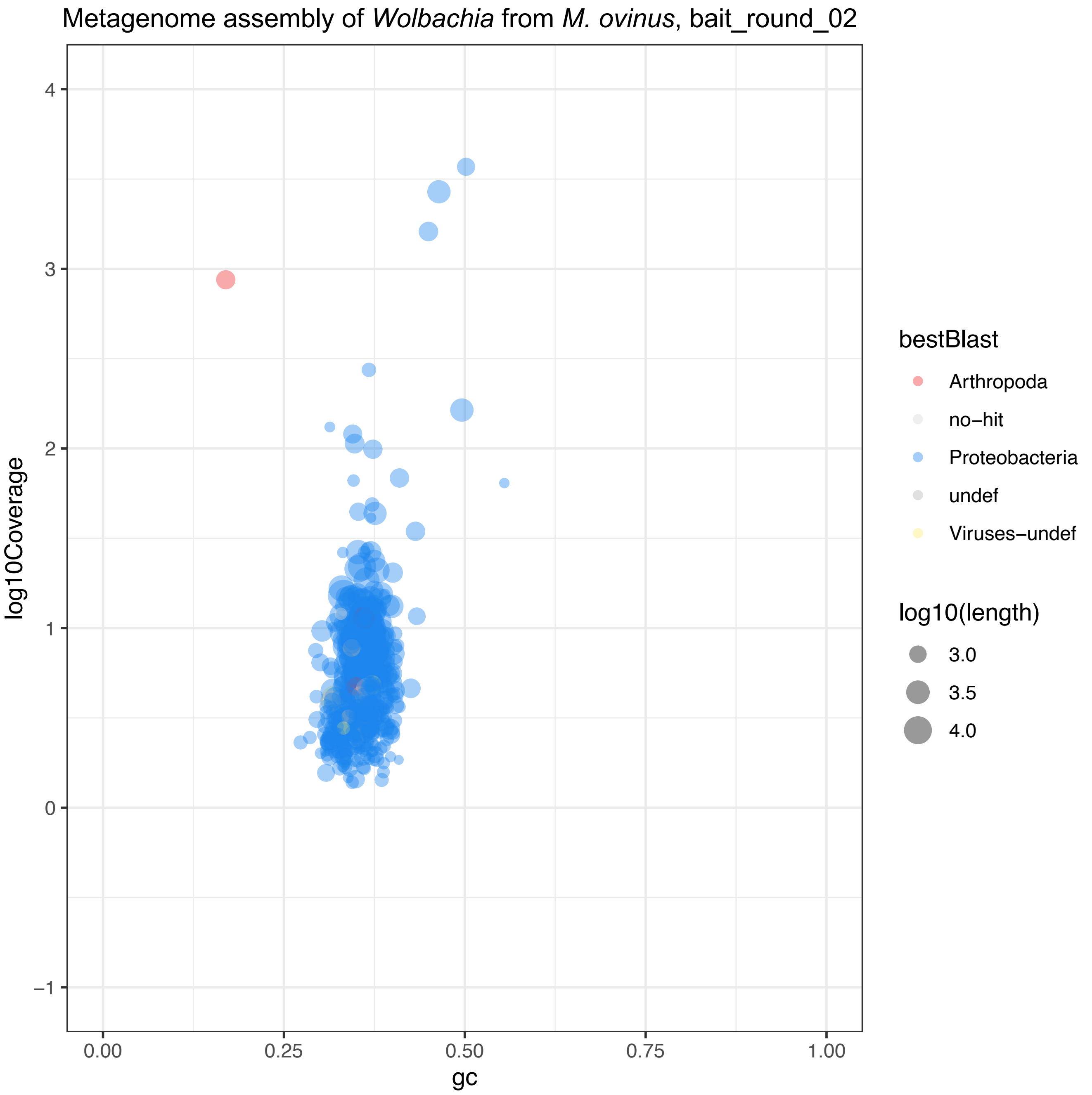

Figure S8(b)

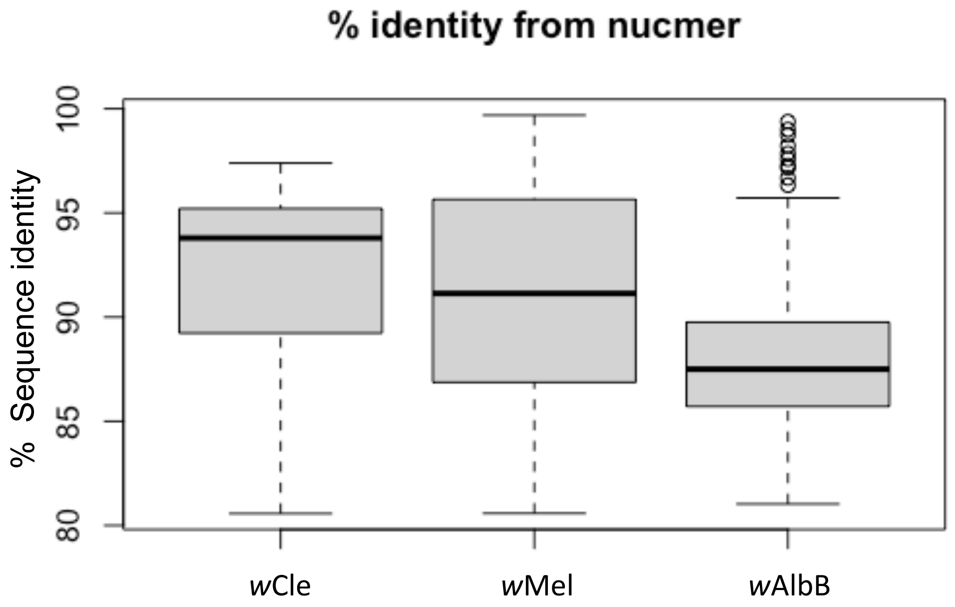

Figure S8(c)

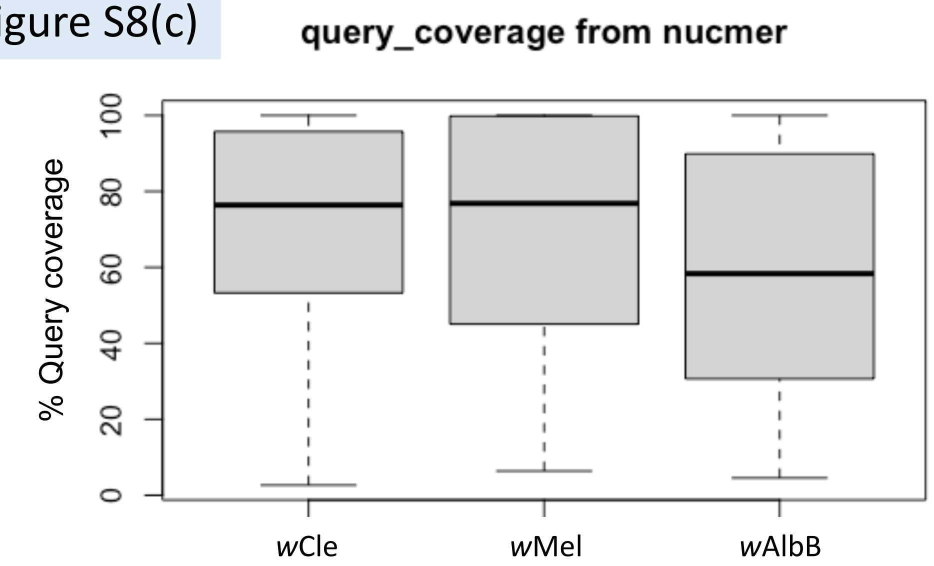

Figure S8(d)

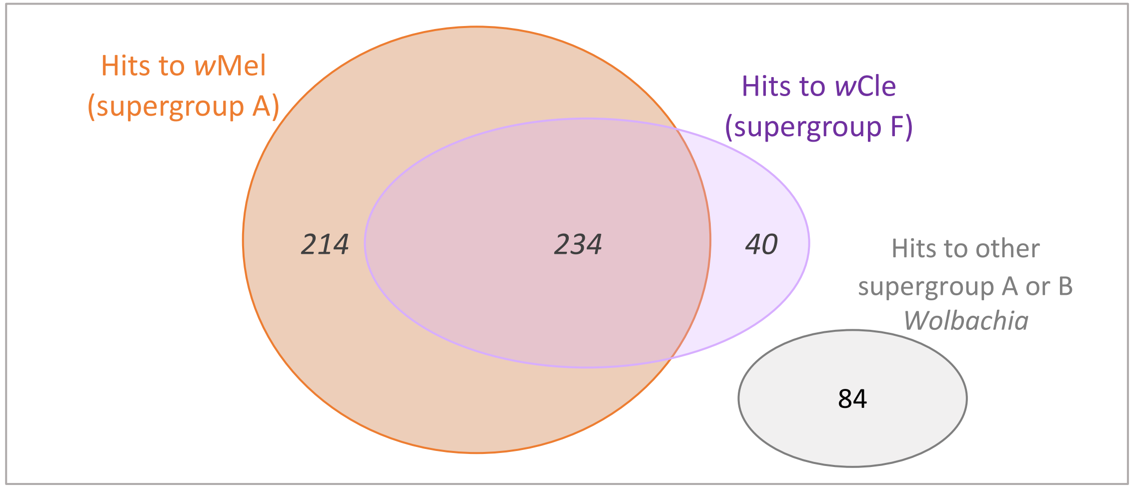

Figure S8(e)

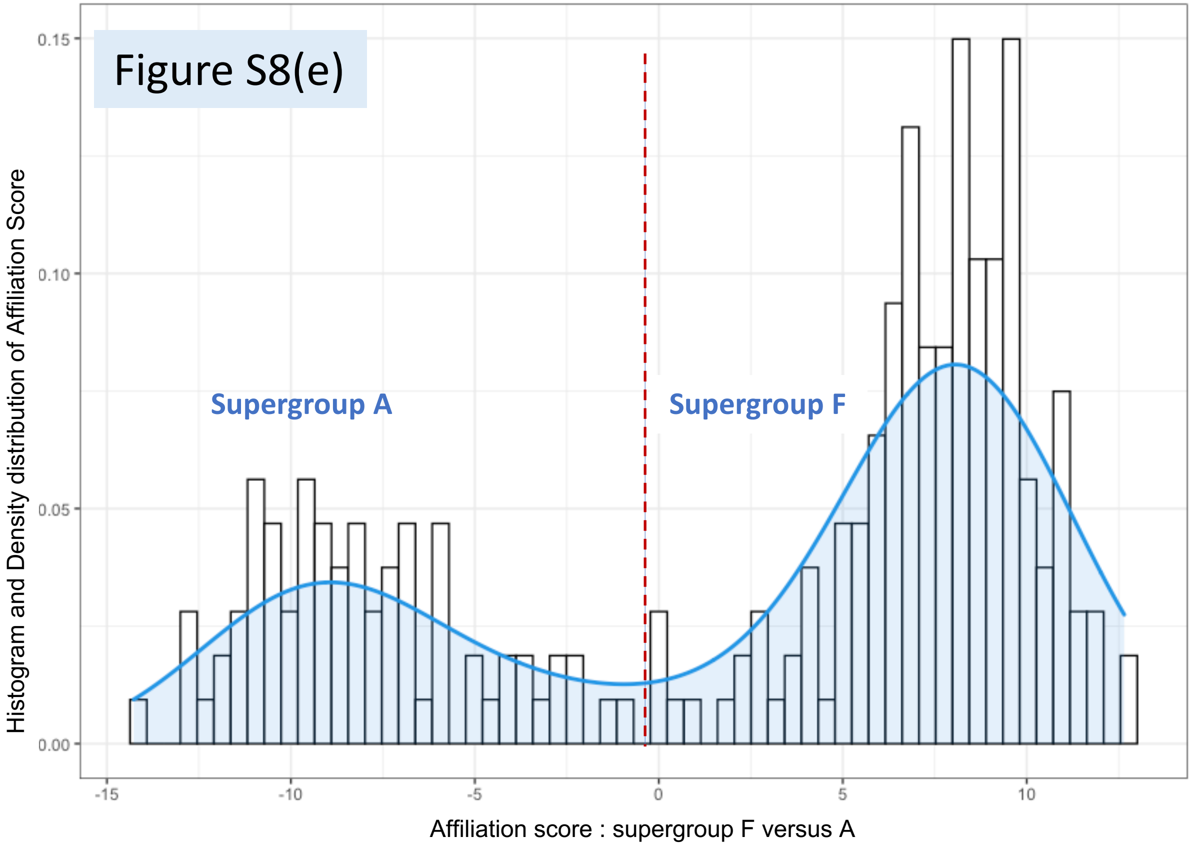

Figure S8(f)

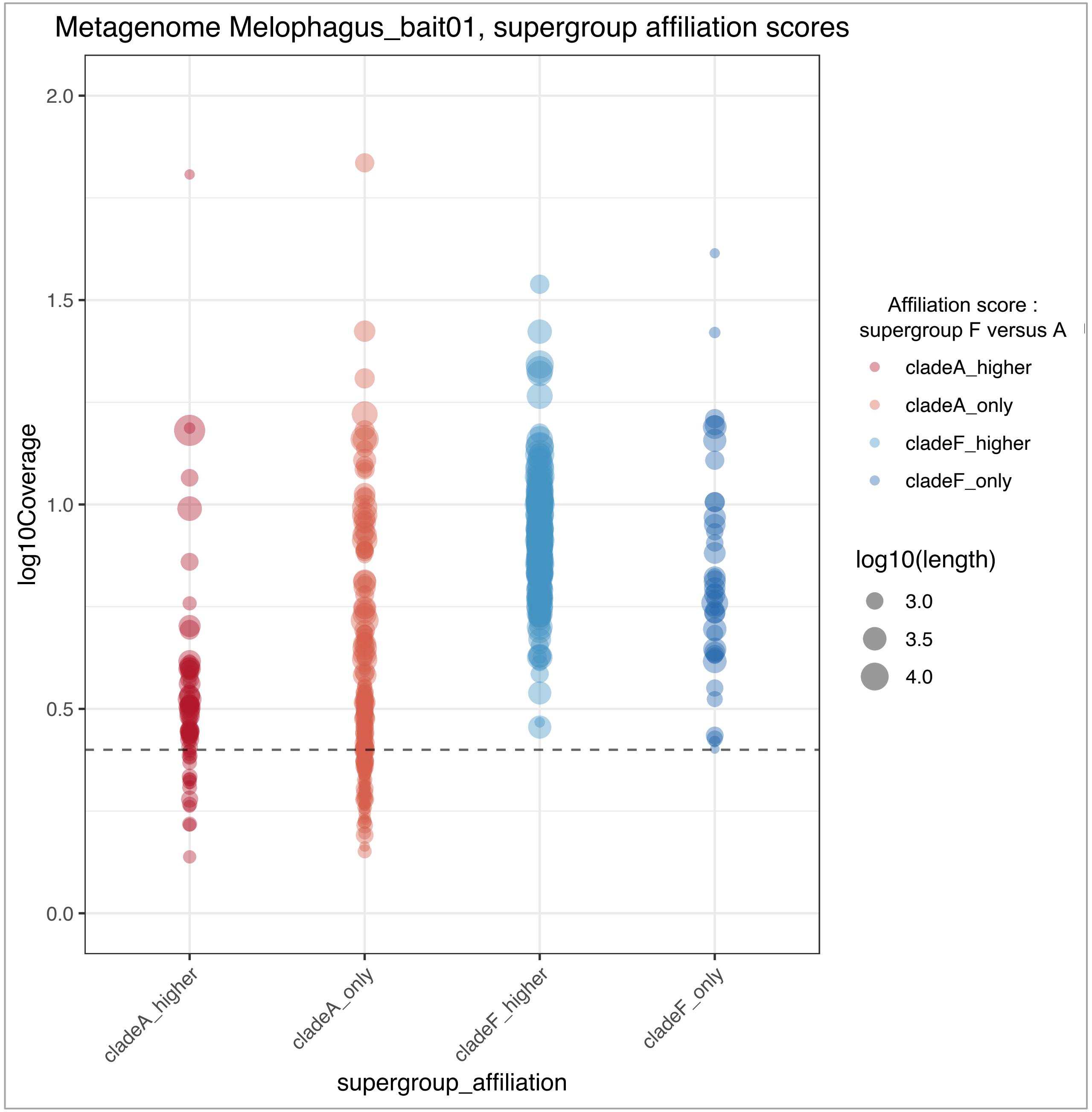

Figure S8(g)

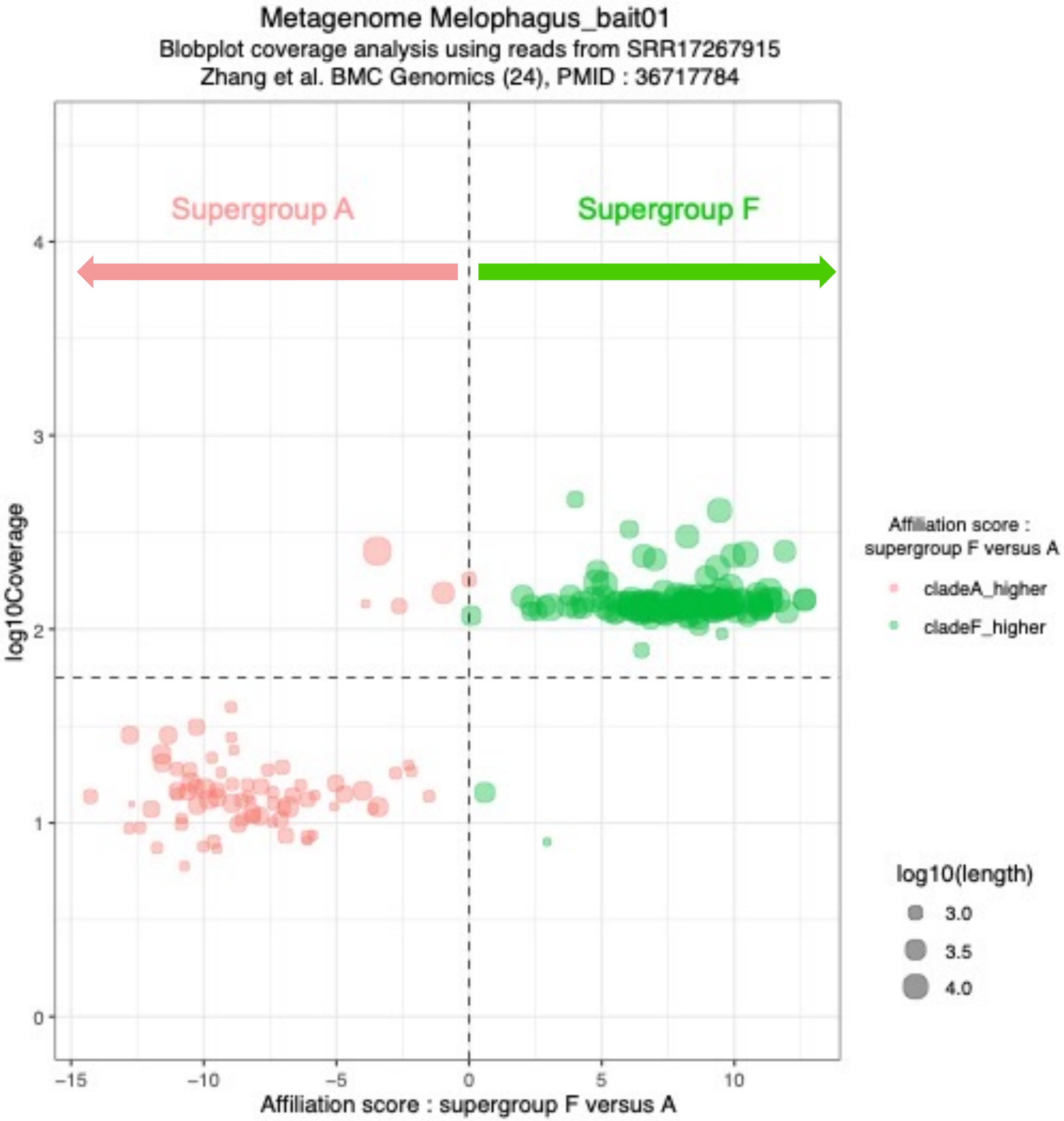

Metagenome Melophagus_bait01
Blobplot coverage analysis using reads from SRR17267915
Zhang et al. BMC Genomics (24), PMID : 36717784
Supergroup A
Supergroup F
log10Coverage
Affiliation score : supergroup F versus A
Affiliation score : supergroup F versus A
cladeA_higher
cladeF_higher
log10(length)
3.0
3.5
4.0

Figure S8(h)

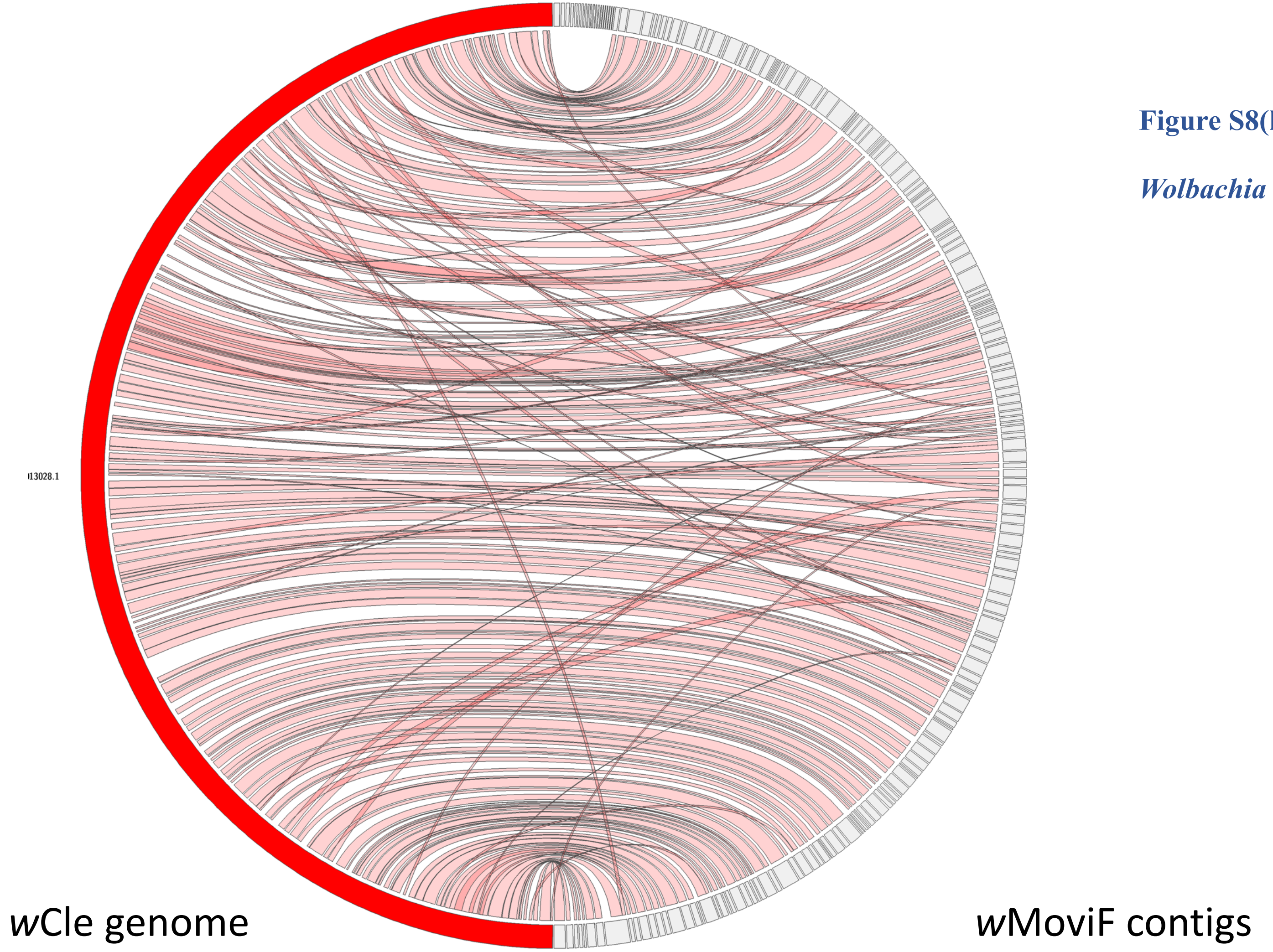

Figure S8(h). Jupiter plot representation of whole-genome alignment between the *Wolbachia w*MoviF assembly versus the complete reference genome of *w*Cle

Figure S9. BlobPlot for assembly WOLB1015 shows *Wolbachia* contigs spread over an unusually wide range of read-coverage, from 1X to 1,000X, indicating the presence of multiple *Wolbachia* in this assembly.

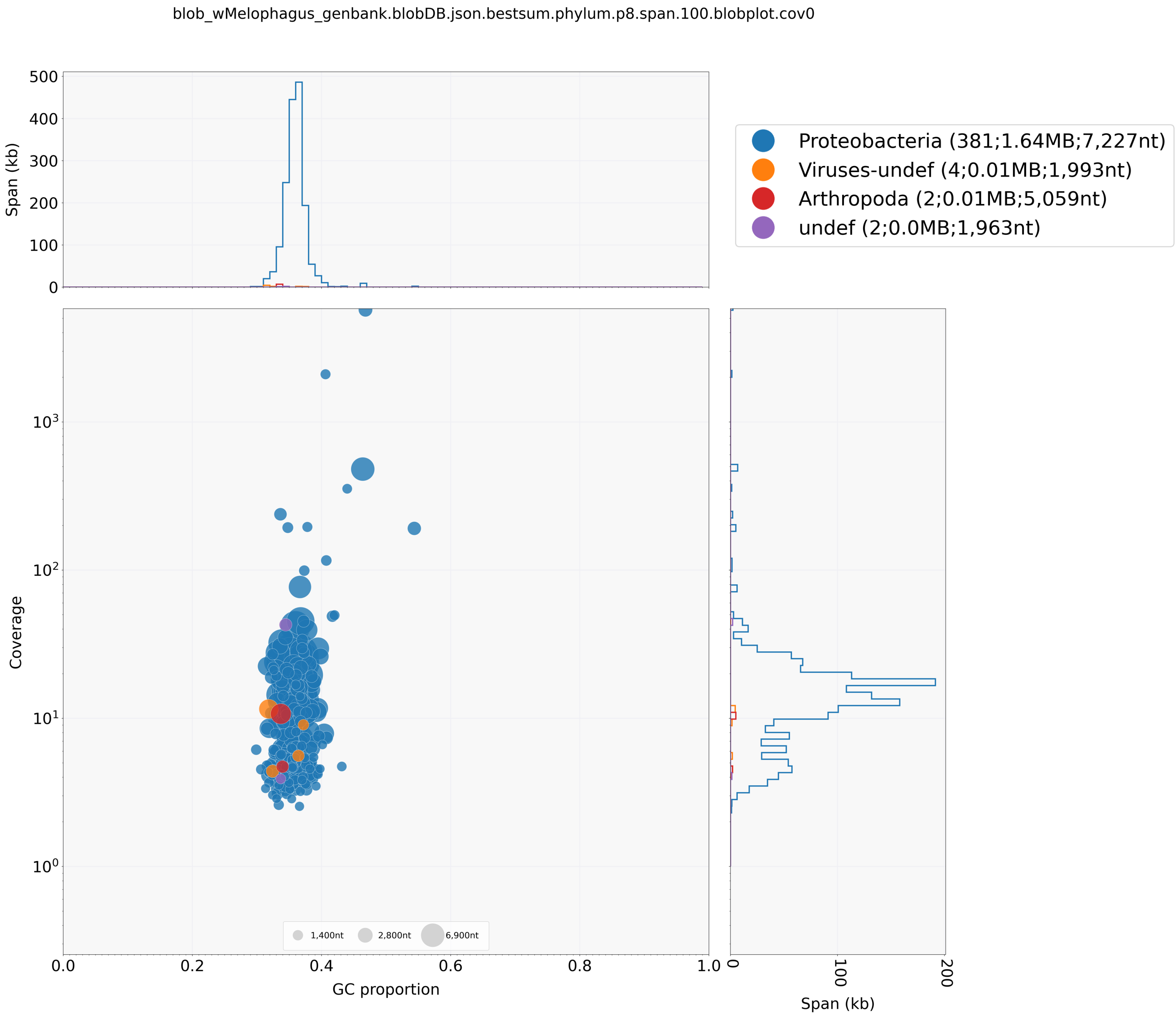

Figure S10. Global comparisons between multiple *Wolbachia* genomes using (a) genome-wide Average Nucleotide Identity (gANI) scores, and digital DNA:DNA Hybridization (dDDH) scores between members of (b) supergroup F, (c) supergroup C, and (d) supergroup D.

a

		*w*Ppe	*w*Moz	*w*Mpe	*w*MoviF	*w*Ocae	*w*Cle	*w*Mhie	*w*Lsig	*w*Wb	*w*Bm	*w*Bpa	*w*Ctub	*w*Dcau	*w*Dimm	*w*Oo	*w*Ov
L	*w*Ppe	100															
F	*w*Moz	73	100														
F	*w*Mpe	73	97	100													
F	*w*MoviF	74	92	93	100												
F	*w*Ocae	73	92	92	96	100											
F	*w*Cle	73	92	92	94	93	100										
F	*w*Mhie	73	92	92	94	93	95	100									
D	*w*Lsig	74	83	83	83	83	84	83	100								
D	*w*Wb	74	84	84	84	84	85	85	89	100							
D	*w*Bm	74	83	83	84	84	84	84	89	97	100						
D	*w*Bpa	74	83	83	84	84	84	84	89	97	99	100					
J	*w*Ctub	73	80	80	80	81	81	80	80	80	80	80	100				
J	*w*Dcau	74	80	80	81	81	81	80	80	80	80	80	82	100			
C	*w*Dimm	73	81	81	82	82	82	82	81	81	81	81	83	83	100		
C	*w*Oo	73	82	81	82	82	82	82	81	81	81	82	83	83	86	100	
C	*w*Ov	73	81	82	82	82	82	82	81	81	81	81	83	83	86	99	100

b

	*w*Moz	*w*Mpe	*w*MoviF	*w*Ocae	*w*Cle
*w*Moz					
*w*Mpe	**71.7** [68.7 - 74.5]				
*w*MoviF	47.4 [44.8 - 50]	47.9 [45.3 - 50.5]			
*w*Ocae	45.4 [42.9 - 48]	46.3 [43.7 - 48.8]	66.6 [63.6 - 69.4]		
*w*Cle	43.6 [41.1 - 46.2]	44.1 [41.5 - 46.6]	53.9 [51.2 - 56.6]	50.0 [47.4 - 52.6]	
*w*Mhie	45.3 [42.7 - 47.9]	45.7 [43.1 - 48.2]	54.1 [51.4 - 56.8]	52.3 [49.7 - 55]	61.8 [58.9 - 64.6]

c

	*w*Oo	*w*Ov
*w*Oo		
*w*Ov	**96.1** [94.8 - 97.2]	
*w*Dimm	29.2 [26.8 - 31.7]	29.1 [26.7 - 31.6]

d

	*w*Bm	*w*Bp	*w*Wb
*w*Bm			
*w*Bp	**93.3** [91.4 - 94.8]		
*w*Wb	70.7 [67.7 - 73.5]	70.3 [67.3 - 73.1]	
*w*Lsig	36.5 [34 - 39]	36.4 [33.9 - 38.9]	36.8 [34.3 - 39.3]

(a) gANI scores were calculated between pairs of *Wolbachia* from filarial and plant-parasitic nematodes, as well as the arthropod *Wolbachia w*Cle. Hierarchical clustering of pairwise gANI scores, presented as a correlation matrix, ordered the various *Wolbachia* in a pattern recapitulating their supergroup assignments. The red lines mark the boundaries between clusters representing different supergroups. dDDH scores between members of (b) supergroup F (c) supergroup C, and (d) supergroup D.

Figure S11. No evidence for biotin biosynthesis genes in any supergroup F *Wolbachia* except *w*Cle

Raw sequencing reads from various supergroup group F *Wolbachia* do not show any alignments within biotin operon of *w*Cle, even though they have alignments mapping to the flanking regions.

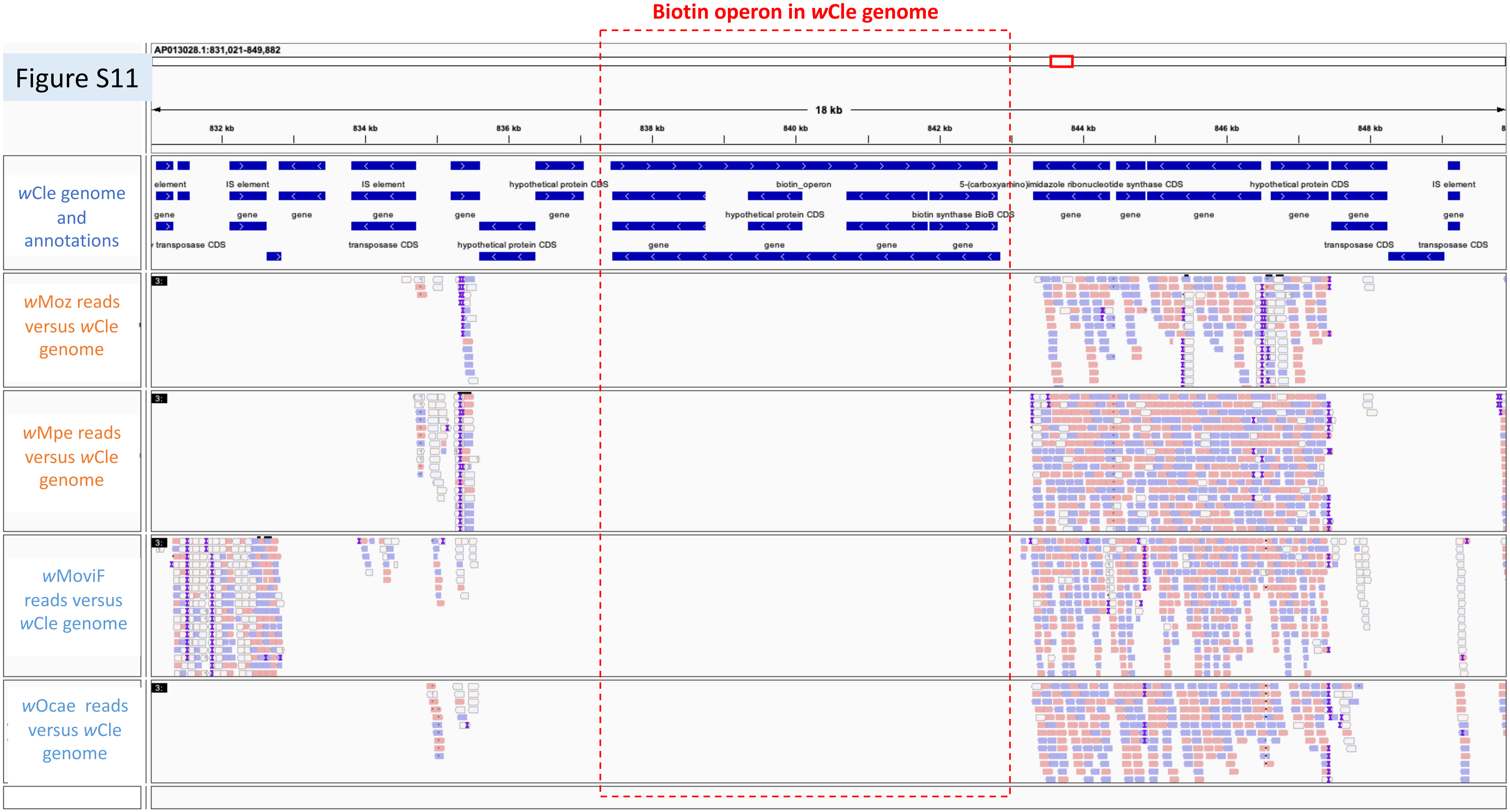

Figure S12

<u>Phylogenomic tree of 127 *Wolbachia* genomes based on 46 Single Copy Orthologs conserved across all *Wolbachia*</u>

The tree was generated by the OrthoFinder software using default parameters.

Total amino acids in the concatenated supermatrix = 8,394

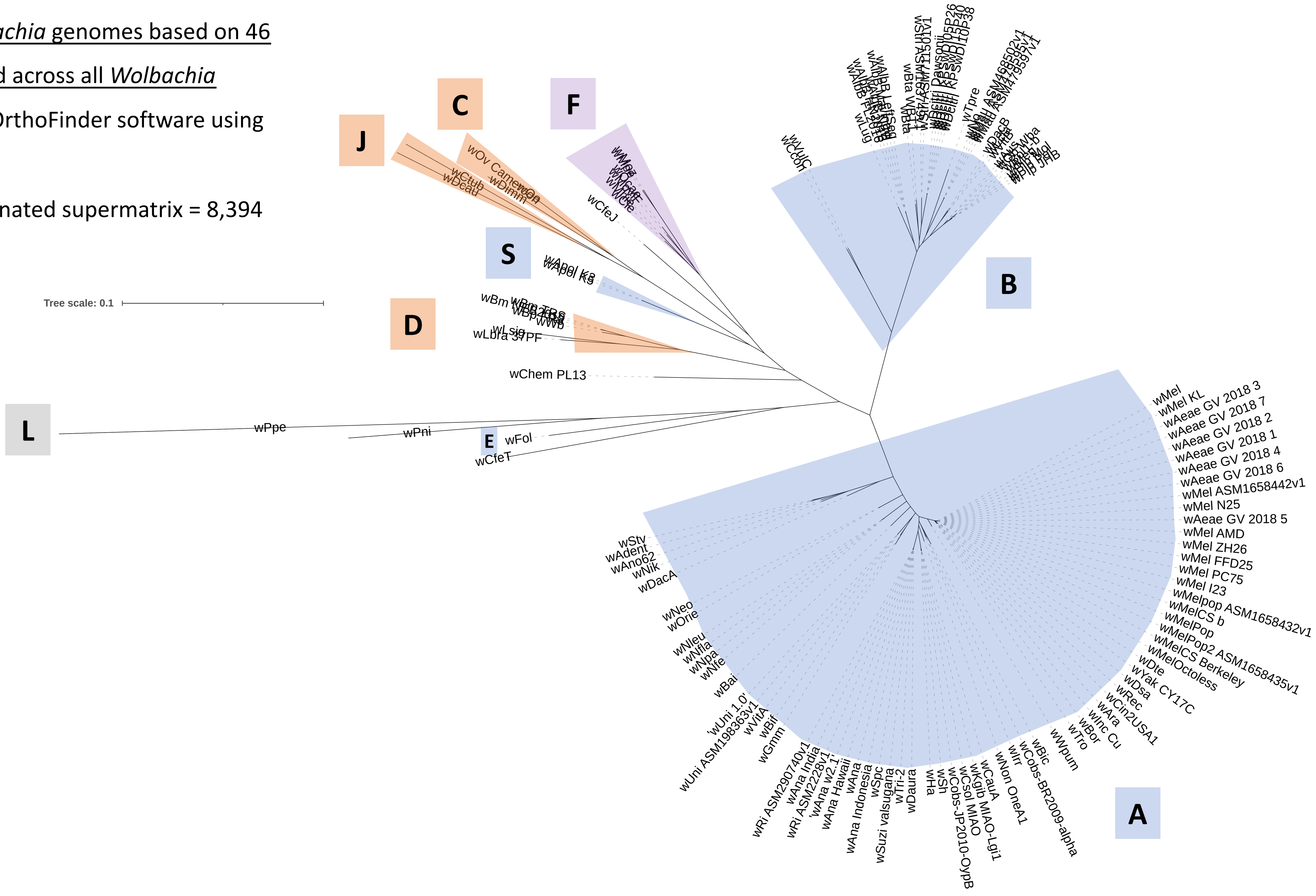

Figure S13

Phylogenomic tree of 81 *Wolbachia* genomes based on 99 Single Copy Orthologs

Total amino acids in the concatenated supermatrix = 20,470

Substitution model selection was performed ModelFinder applied to each gene separately using a partition model.

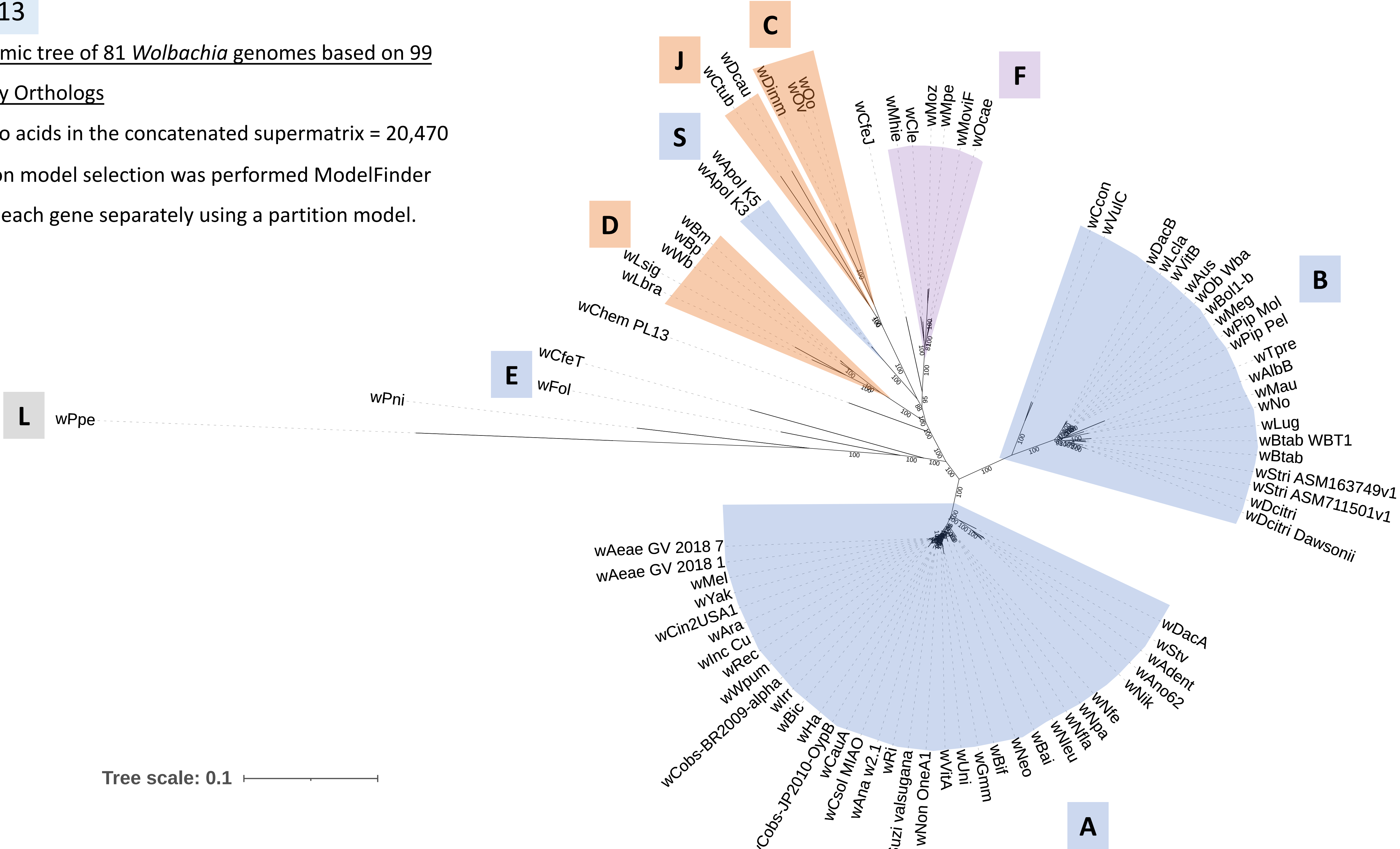

Figure S15(a)

Multiple sequence alignment of the predicted protein / ORF of the *bfr* region across all filarial *Wolbachia*, with the arthropod bacterioferritin protein sequences included as references for a complete ORF. Premature stop codons in each amino acid sequence are shown as asterisks.

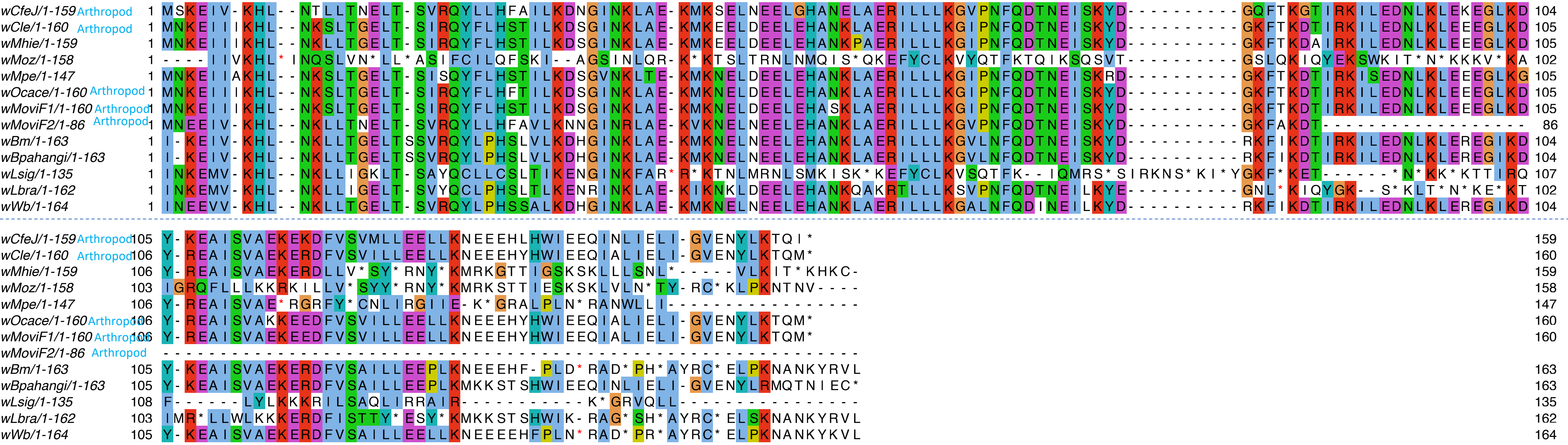

Figure S14(b)

Multiple sequence alignment of the DNA sequence in the *bfr* gene region for all filarial *Wolbachia*, with arthropod *Wolbachia w*Cle, *w*Ocae, *w*MoviF and *w*CfeJ sequences as references for an intact *bfr* gene.

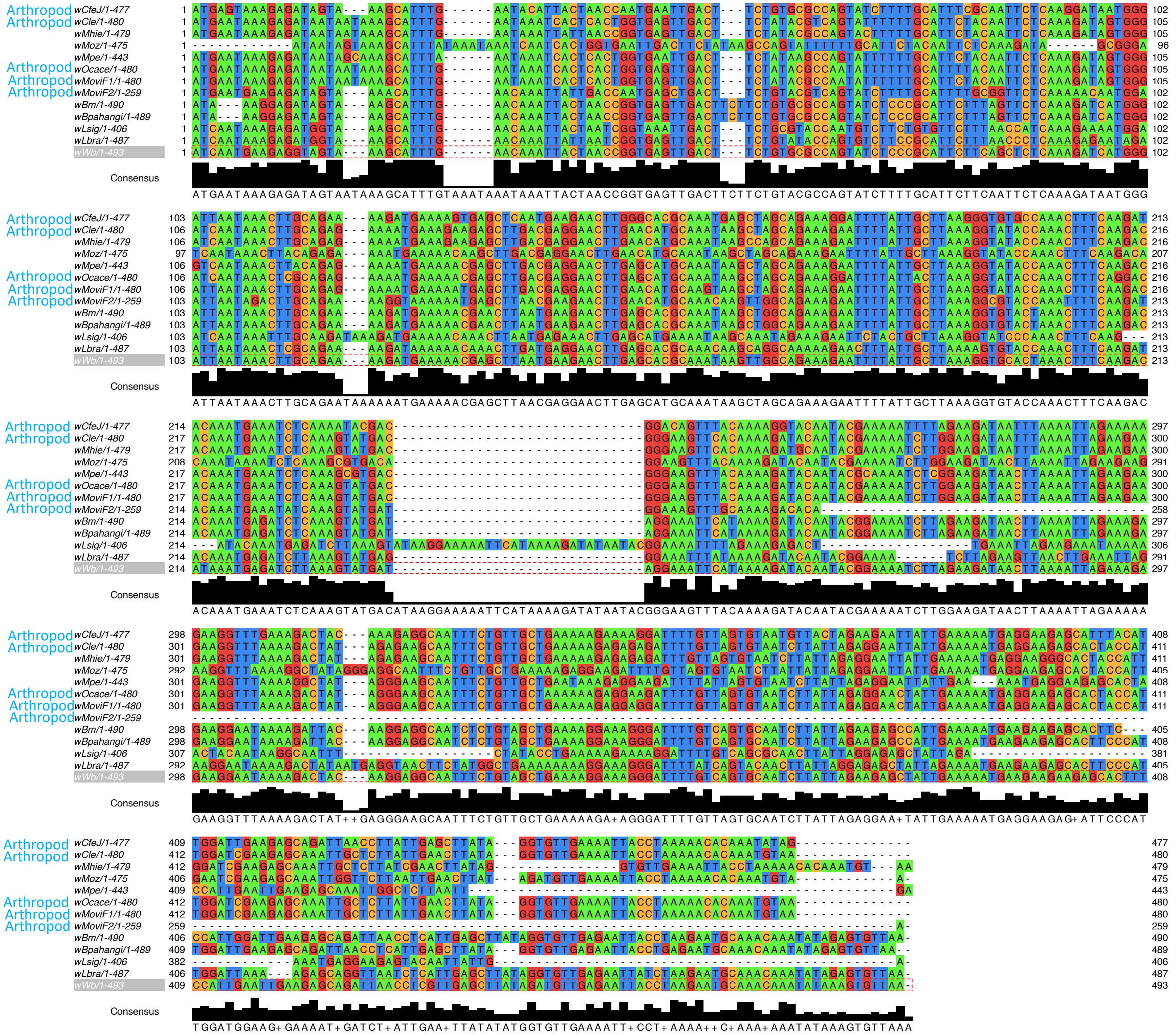

Figure S14(c)

Multiple sequence alignment of the genomic region flanking the bacterioferritin gene *bfr* shows conserved syntenic genes.

The sequences are grouped according to their supergroup. Arthropod *Wolbachia* sequences are labelled as such. Annotated *bfr* pseudogenes are highlighted in magenta. Regions of tblstn hit to a particular *Wolbachia* genome are indicated in purple arrows. No tblastn hit could be found in the genome of the filarial *Wolbachia w*Dcau, even though the rest of the genomic region was syntenic with other *Wolbachia*.

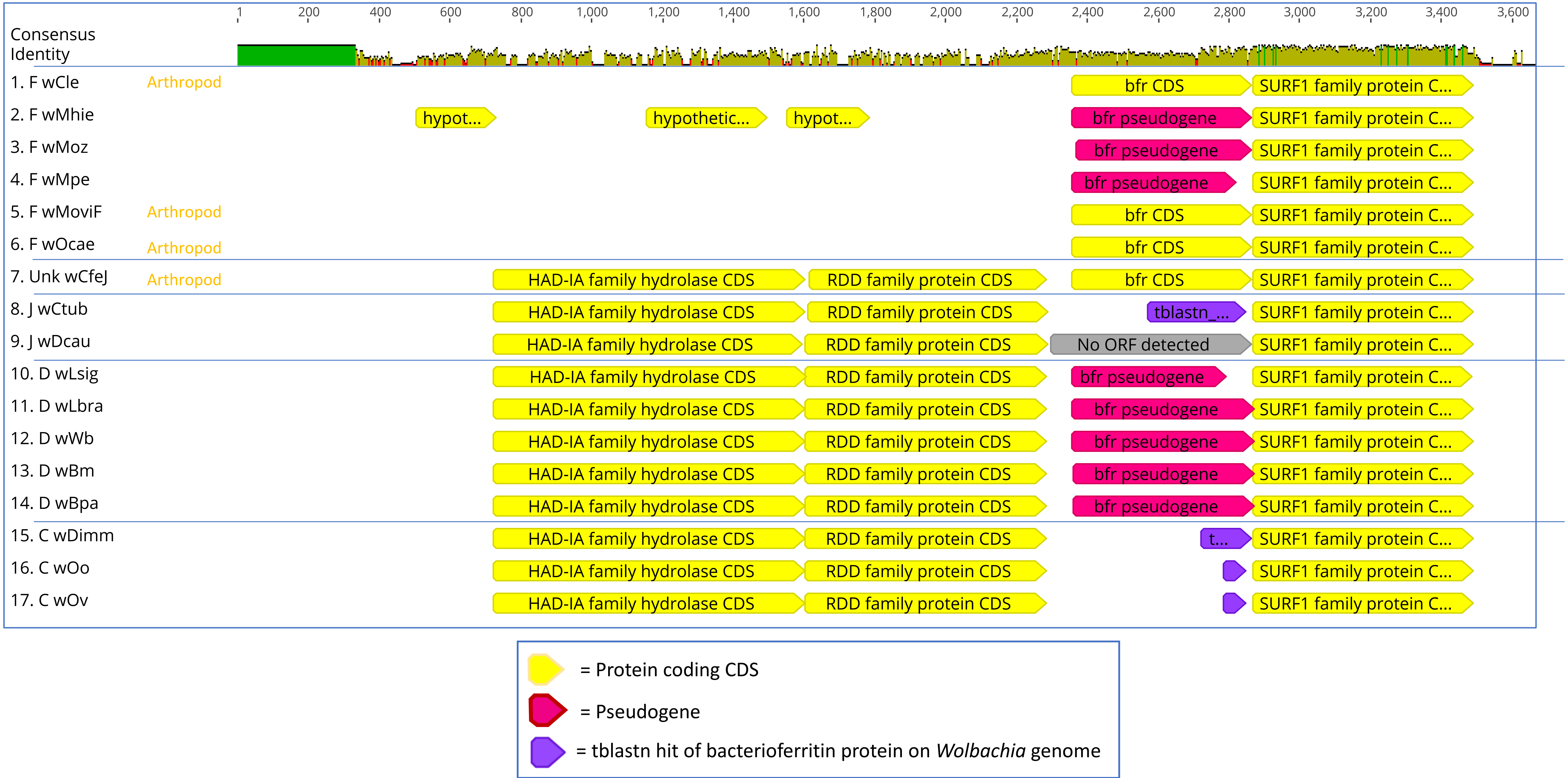

Figure S15 (a) Results from Xia's test for substation saturation indicate little to no saturation (Iss < Iss.c, pvalue = 0) in *bfr* gene sequence across *Wolbachia*.
(b) Transitions (Ts) and transversions (Tv) versus genetic distance (F84 metric) form *bfr* genes across *Wolbachia* also indicate that Ts and Tv increase linearly with genetic distance and are not saturated.
Gene sequences from the following Wolbachia were analyzed: *w*Cle, *w*MoviF, *w*Ocae (supergroup F), *w*Apol_K3 (supergroup S), *w*Mel and *w*DacA (supergroup A), *w*AlbB and *w*Vulc (supergroup B), and *w*CfeJ, *w*ChemPL13, *w*Pni, *w*CfeT and *w*Fol.

Data Analysis in Molecular Biology

File Edit Alignment Sequences Seq. Analysis Phylogenetics Structure Bioinfo Genomics Graphics Tools

```
Test of substitution saturation (Xia et al. 2003; Xia and Lemey 2009)

Analysis performed on fully resolved sites only.

Testing whether the observed Iss is significantly
lower than Iss.c.

Part I. For a symmetrical tree.
=========================================================
Prop. invar. sites                  0.1750
Mean H                              0.2760
Standard Error                      0.0229
Hmax                                1.6827
Iss                                 0.1640
Iss.c                               0.6897
T                                  22.9513
DF                                261
Prob (Two-tailed)                   0.0000
95% Lower Limit                     0.1190
95% Upper Limit                     0.2091

=========================================================
Part II. For an extreme asymmetrical (and generally very
unlikely) tree.
=========================================================
Iss.c                               0.5134
T                                  15.2537
DF                                261
Prob (Two-tailed)                   0.0000

95% Lower Limit                     0.1190
95% Upper Limit                     0.2091

=========================================================

Interpretation of results:
               Significant Difference
               ----------------------
               Yes                 No
--------------------------------------------------------
Iss < Iss.c    Little              Substantial
               saturation          saturation
--------------------------------------------------------
Iss > Iss.c    Useless             Very poor
               sequences           for phylogenetics
--------------------------------------------------------
```

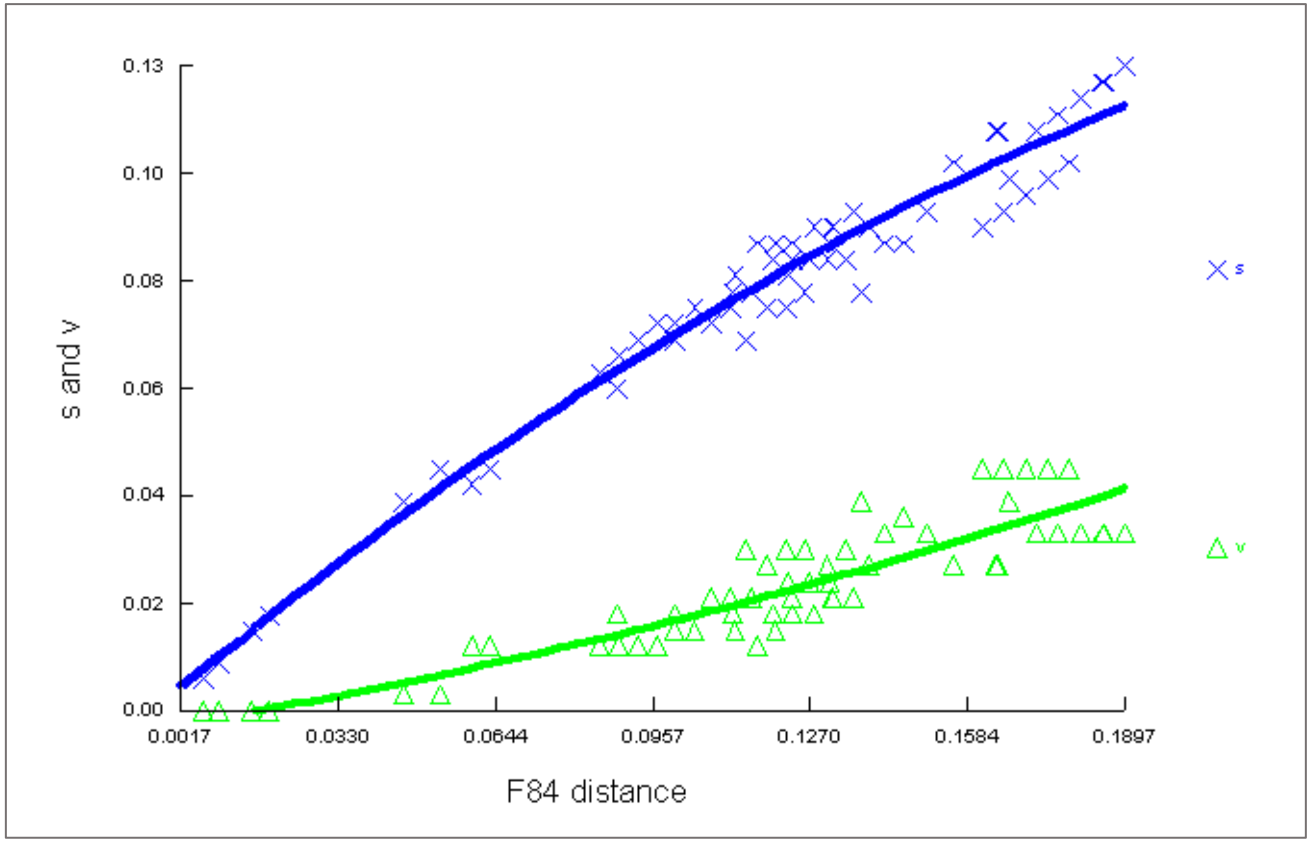

Figure S16

dN/dS values for bacterioferritin locus in *w*Bm and *w*Bpa, calculated in comparison to the reference sequence in *w*ChemPL13.

The density plot shows the distribution of dN/dS values for all potential pseudogenes detected by Pseudofinder. The median values are marked by dotted black lines, and the values for bfr loci are marked in dotted red lines.

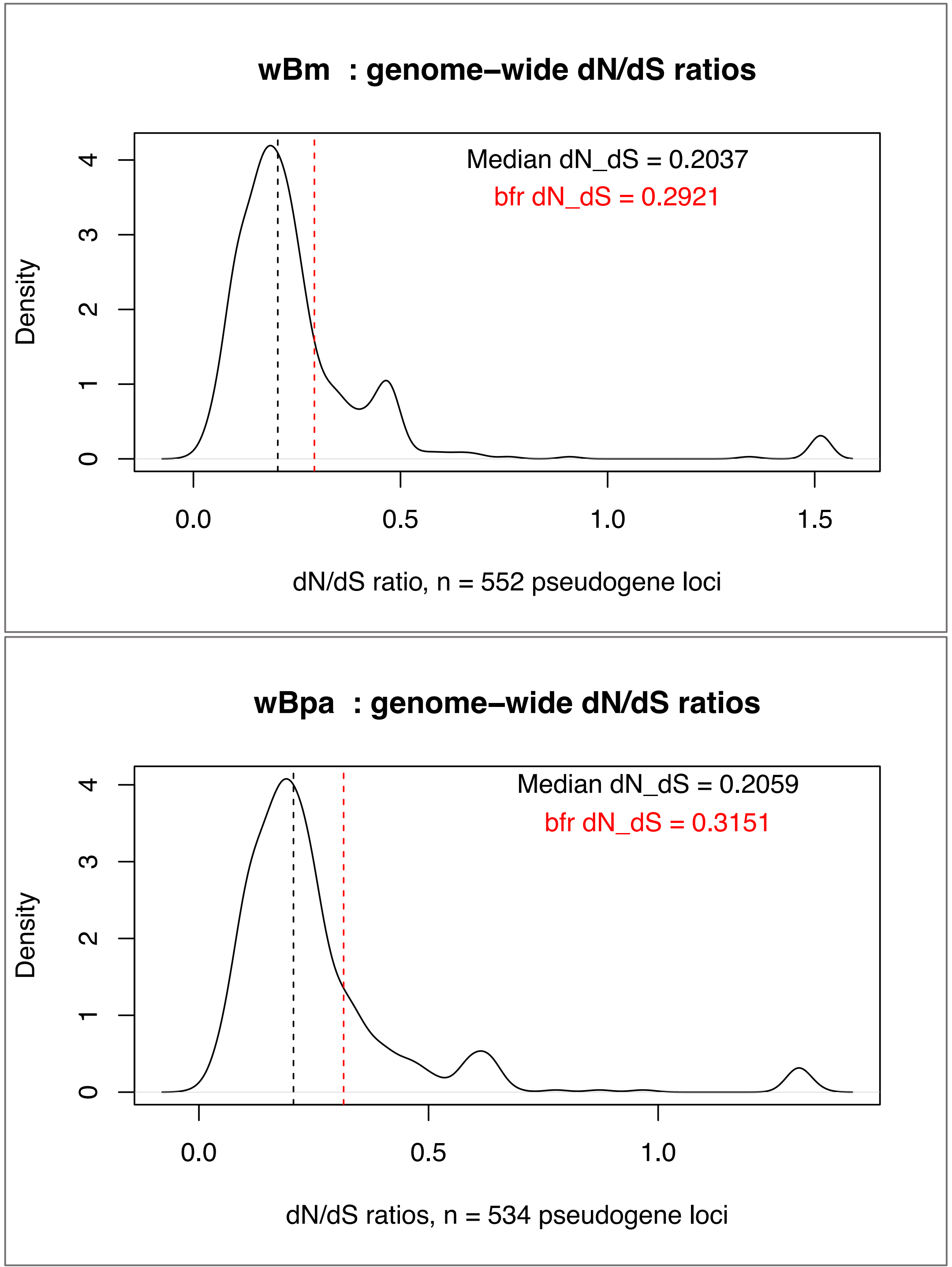

Supplement: evad073_Supplementary_Data [file evad073_supplementary_data.zip › Supp-Figures.v10.pdf]
